# Single-Atom Catalysts for Selective Oxygen Reduction: Transition Metals in Uniform Carbon Nanospheres with High Loadings

**DOI:** 10.1021/jacsau.3c00557

**Published:** 2023-10-19

**Authors:** Jacob Jeskey, Yong Ding, Yidan Chen, Zachary D. Hood, George E. Sterbinsky, Mietek Jaroniec, Younan Xia

**Affiliations:** † School of Chemistry and Biochemistry, Georgia Institute of Technology, Atlanta, Georgia 30332, United States; ‡ School of Materials Science and Engineering, Georgia Institute of Technology, Atlanta, Georgia 30332, United States; § Applied Materials Division, Argonne National Laboratory, Lemont, Illinois 60439, United States; ∥ Advanced Photon Source, Argonne National Laboratory, Lemont, Illinois 60439, United States; ⊥ Department of Chemistry and Biochemistry, Kent State University, Kent, Ohio 44242, United States; # The Wallace H. Coulter Department of Biomedical Engineering, Georgia Institute of Technology and Emory University, Atlanta, Georgia 30332, United States; ■ School of Chemical and Biomolecular Engineering, Georgia Institute of Technology, Atlanta, Georgia 30332, United States

**Keywords:** single-atom catalyst, carbon nanospheres, chemical confinement, one-pot, oxygen reduction reaction

## Abstract

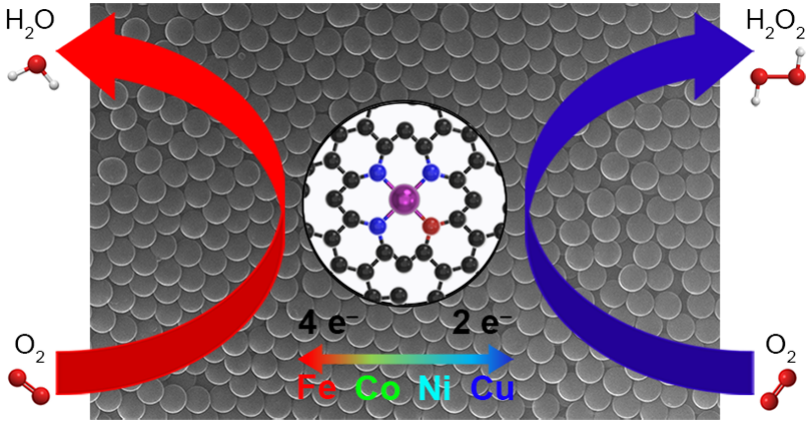

Transition metal single-atom catalysts (SACs) in uniform carbon nanospheres have gained tremendous interest as electrocatalysts owing to their low cost, high activity, and excellent selectivity. However, their preparation typically involves complicated multistep processes that are not practical for industrial use. Herein, we report a facile one-pot method to produce atomically isolated metal atoms with high loadings in uniform carbon nanospheres without any templates or postsynthesis modifications. Specifically, we use a chemical confinement strategy to suppress the formation of metal nanoparticles by introducing ethylenediaminetetraacetic acid (EDTA) as a molecular barrier to spatially isolate the metal atoms and thus generate SACs. To demonstrate the versatility of this synthetic method, we produced SACs from multiple transition metals, including Fe, Co, Cu, and Ni, with loadings as high as 3.87 wt %. Among these catalytic materials, the Fe-based SACs showed remarkable catalytic activity toward the oxygen reduction reaction (ORR), achieving an onset and half-wave potential of 1.00 and 0.831 *V*_RHE_, respectively, comparable to that of commercial 20 wt % Pt/C. Significantly, we were able to steer the ORR selectivity toward either energy generation or hydrogen peroxide production by simply changing the transition metal in the EDTA-based precursor.

## Introduction

With the global environmental crisis and ever-increasing demands on energy, development of sustainable systems for energy conversion has become one of the most important challenges today.^1,2^ Currently, electrochemical energy conversion technologies, including fuel cells, water splitting, and metal–air batteries, are considered the most promising means to meet the increasing requirements on energy due to their high-energy densities, high efficiency, and environmental greenness.^3−5^ Unfortunately, their practical use is challenged by the high cost, sluggish kinetics, and long-term stability issues resulting from the use of noble-metal electrocatalysts.^6^ It is of great importance to design and develop alternative catalytic materials and systems using earth-abundant, cost-effective, and sustainable elements.

Recently, single-atom catalysts (SACs) anchored to carbon nanosphere supports have gained tremendous attention as possible alternatives for noble-metal nanoparticles.^7,8^ Unlike noble-metal nanoparticles, which often contain over 80% bulk-phase atoms (i*.*e., atoms inaccessible to the reactants), atomically dispersed metal atoms exhibit total atom utilization, maximizing mass activity while effectively reducing the material cost.^9^ Moreover, the inherent undercoordination of atomically dispersed metal atoms results in the formation of dangling bonds (i*.*e*.*, unsaturated valence), which play an important role in the adsorption and activation of reactants on catalytic sites.^7,10,11^ As such, reducing to atomic scale can alter the reaction thermodynamics, leading to unprecedented activity and selectivity toward various reactions.^12−14^ Meanwhile, the carbon nanosphere support is responsible for maintaining atomic isolation and ensuring sufficient mass diffusion and conductivity during electrocatalysis. Unfortunately, most of the synthetic approaches are plagued by a trade-off between metal loading and the morphology of the carbon nanospheres. By far the most common approach to produce SACs on carbon nanospheres is the use of incipient wetness impregnation (IWI), which introduces the metal species onto preprepared substrates by means of adsorption. Even though the carbon nanosphere morphology is typically preserved, these multistep techniques are not practical for industrial applications as they suffer from extremely low loadings (often, below 0.6 wt %) and are expensive and time-consuming.^15−18^ Alternatively, one-pot methods, where the metal species are introduced during the synthesis of carbon nanospheres, are much simpler and can lead to higher SAC loadings, albeit the carbon support may take irregular morphologies and formation of metal nanoparticles still remains an issue.^18−20^

In general, the main problem with a one-pot method appears to stem from the incompatibility between the SAC precursor (typically acidic metal salts) and the alkaline environment involved in the synthesis. Typically, the phenolic resin achieves its spherical morphology when the reactants first polymerize into nanodroplets, followed by the acquisition of surface charges to help prevent aggregation.^21^ However, when acidic metal salts are added during the initial polymerization, they react with the available base to form undesirable metal hydroxide nanoparticles, which disrupt the polymerization process and impact the spherical morphology. These hydroxide nanoparticles have to be removed in the subsequent steps using a strong acid, which also removes a significant portion of SACs while increasing the complexity of the production process.^20^ In principle, these issues can be addressed by switching to metal complexes that can withstand an alkaline environment. Specifically, one can turn to a chemical confinement strategy, where the metal ions are first bound to a strong chelating ligand to protect them from the reaction environment and thus suppress the formation of nanoparticles. In our previous work, we found that ethylenediaminetetraacetic acid (EDTA) can be readily incorporated into the emulsion polymerization between 3-aminophenol and formaldehyde to produce N-doped carbon nanospheres with uniform and tunable sizes.^22^ Since EDTA is a hexadentate ligand capable of forming stable complexes with most transition metals, it makes sense that it can also be used to protect the metal ions while maintaining high uniformity. Meanwhile, the resulting cage-like coordination of metal-EDTA complexes may act as a molecular barrier to spatially isolate the resultant metal atoms.

Herein, we report a general one-pot strategy to produce transition metal SACs in uniform carbon nanospheres with high metal loadings up to 3.87 wt % by leveraging the power of ligand confinement. The synthesis was demonstrated to be robust as multiple transition metals (e.g., Fe, Cu, Co, and Ni) can be readily incorporated in high loadings without formation of nanoparticles. Moreover, owing to their high loadings and uniform morphology, the SAC-loaded carbon nanospheres exhibited excellent catalytic activity toward the oxygen reduction reaction (ORR).

## Experimental Section

### Chemicals and Materials

3-Aminophenol, formaldehyde (37 wt %), ammonium hydroxide (28–30 wt %), iron(III) chloride hexahydrate, cobalt(II) chloride hexahydrate, copper(II) chloride dihydrate, nickel(II) chloride hexahydrate, disodium ethylenediaminetetraacetate dihydrate (EDTA), and Nafion (5 wt %) were all purchased from Sigma-Aldrich. Ethanol (200 proof) and sodium hydroxide were ordered from VWR. All aqueous solutions were prepared by using deionized (DI) water with a resistivity of 18.2 MΩ·cm at room temperature.

### Synthesis of Metal-EDTA (M-EDTA) Complex

Typically, 3.72 g of EDTA was dissolved in 10.0 mL of 1.0 M sodium hydroxide and heated to 80 °C. Next, 0.09 mol of the desired metal salt (e.g., iron chloride, cobalt chloride, copper chloride, or nickel chloride) was dissolved in 5.0 mL of water and then added to the EDTA solution. The solution was heated to 80 °C for 24 h and then boiled until a precipitate occurred. After the solution cooled to room temperature, the crystals were filtered and washed with cold water and ethanol.

### Metal-EDTA-Mediated Synthesis of Phenolic Resin Nanospheres

In a typical synthesis, 0.6 g of 3-aminophenol was dissolved in a solution containing 40 mL of water and 16 mL of ethanol. Then, 0.15 g of the metal-EDTA precursor was added, followed by the addition of 0.25 mL of ammonium hydroxide to raise the pH to 9.25. Finally, 0.36 mL of 37 wt % formaldehyde was added dropwise, and the mixture was stirred for 4 h at room temperature. The as-obtained mixture was transferred into a 125 mL Teflon container and subjected to thermal treatment at 80 °C for 20 h. The resulting polymer product was collected by centrifugation at 11 000 rpm for 15 min.

### Carbonization and In Situ Reduction

Carbon nanospheres loaded with transition metal SACs were prepared by heating the sample in a tube furnace under flowing N_2_ up to a final temperature of 1000 °C at a heating rate of 2 °C min^–1^ for 2 h. The carbon nanospheres loaded with SACs were termed CS-X-E, where X-E refers to the metal-EDTA complex used.

### Characterizations

High-angle annular dark-field scanning TEM (HAADF-STEM) and energy-dispersive X-ray (EDX) mapping images were acquired using an aberration-corrected Hitachi HD-2700 STEM. Transmission electron microscopy (TEM) images were obtained on a Hitachi HT7700. Prior to TEM analysis, the sample was dispersed in ethanol by moderate sonication, followed by deposition on a lacey carbon coated, 200 mesh copper TEM grid by drop-casting, followed by drying under ambient conditions. Scanning electron microscopy (SEM) images were obtained by using a Hitachi SU-8230 microscope. Prior to SEM analysis, samples were dispersed in ethanol by moderate sonication, then deposited on silicon wafers, and dried under ambient conditions. Thermogravimetric analysis (TGA) was conducted on a SDT Q-600 analyzer up to 800 °C under air, with a heating rate of 10 °C min^–1^ using high-resolution SDT mode. The initial weight of each sample was in the range of 15–20 mg. X-ray photoelectron spectroscopy (XPS) data were collected on a Thermo K-Alpha spectrometer with an Al Kα source. X-ray absorption spectroscopy (XAS) experiments on the Fe K-edge, Co K-edge, Cu K-edge, and Ni K-edge were conducted in fluorescence mode at beamline 9-BM of the Advanced Photon Source (APS) at Argonne National Laboratory. The data obtained from XAS were processed and analyzed using the ATHENA program.^23^ The average valence state of each SAC species was derived from the K-edge absorption threshold. The least-squares EXAFS fitting data was configured from the coordination route of known metal nitride and oxide references using the ARTEMIS program.^23^

### Catalytic Measurements

The electrochemical measurements were conducted at room temperature using a three-electrode cell and a WaveDriver 200 EIS Bipotentiostat electrochemical workstation. A rotating ring-disk electrode (RRDE, 5 mm in diameter) loaded with the catalyst served as the working electrode together with a Pt wire in a fritted isolation tube as the counter electrode and saturated calomel electrode (SCE) as the reference electrode. The working electrode was prepared by polishing with 0.3-μm Al_2_O_3_ slurry and washing with water and ethanol. The working electrode was then polished in the same fashion using 0.05 μm Al_2_O_3_ slurry. The catalyst ink was prepared by ultrasonicating 10 mg of the SAC catalyst with a mixture containing 312.5 μL of H_2_O, 937.5 μL of isopropanol, and 10 μL of 5 wt % Nafion solution for 1 h to form a homogeneous suspension. Afterward, 9 μL of the as-prepared catalyst ink was dropped on the polished RDE and dried at room temperature. As a benchmark, Pt/C catalyst ink (20 wt % Pt nanoparticles on Vulcan XC-72 carbon support, Premetek Co.) was prepared using the same protocol except 1.25 mg of the commercial catalyst was used. Before electrochemical measurements, all solutions were purged and saturated with Ar or O_2_. To measure ORR activity, the catalyst was first cycled 30 times between 0.05 and 1.1 V (vs. reversible hydrogen electrode, RHE) at 100 mV s^–1^ in Ar-saturated 0.1 M KOH. A background CV was obtained under the same conditions, except the scan rate was reduced to 10 mV s^–1^. ORR measurements were conducted by cycling the disk potential 20 times between 0.1 and 1.1 V_RHE_ at 10 mV s^–1^ in O_2_-saturated 0.1 M KOH at 1,600 rpm. The collection efficiency for the RRDE was 25.6% and the ring current was kept constant at 1.2 V_RHE_. Stability measurements were recorded using *i*–*t* chronoamperometry at 0.5 V_RHE_ for 10 h in O_2_-saturated 0.1 M KOH at 1,600 rpm. The ORR plots were corrected for double-layer capacitance by subtracting the background CV scan. All of the electrochemical data were *iR*-compensated at 85%. The onset potential (*E*_onset_) was defined as the potential at which the first derivative starts increasing.

## Results and Discussion

**Figure 1:**
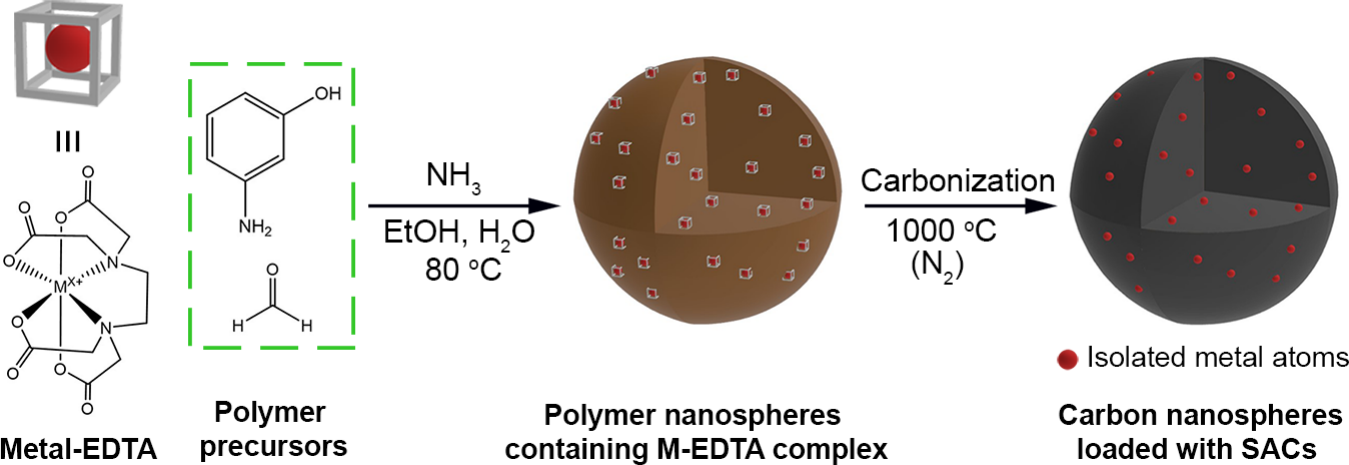
Schematic illustration showing the facile synthesis of carbon nanospheres loaded with transition metal SACs by using a one-pot method.

Previously, we established a facile method to produce uniform carbon nanospheres, where EDTA served as an emulsion stabilizer to prevent the polymer nanospheres from aggregating into irregular structures.^22^ Building upon this work, we found that EDTA could also serve as a transport ligand to load metal ions into the polymer matrix. Figure 1 shows a general strategy for producing SACs in carbon nanospheres by using a one-pot method. During the initial stage of polymerization, 3-aminophenol and formaldehyde react with each other to generate a variety of hydroxymethyl and benzoxazine derivatives, which then form emulsion droplets to minimize the interfacial energy between the hydrophobic oligomers and hydrophilic solution.^24,25^ During this process, the M-EDTA complex is incorporated into the polymer matrix through hydrogen bonding and ionic interactions. The ability to produce SACs at high loadings is largely attributed to the chelating ability of EDTA, which confines an individual metal ion in a cage-like complex while acting as a molecular barrier to spatially isolate the resultant metal atoms from each other in the polymer nanosphere. Moreover, the functional groups in EDTA that bind to the metal ion can also help anchor the metal atom during carbonization and thus prevent the atoms from thermally sintering into nanoparticles. Altogether, once incorporated into the polymer matrix, the EDTA complexes can be decomposed to produce SACs in carbon nanospheres.

**Figure 2:**
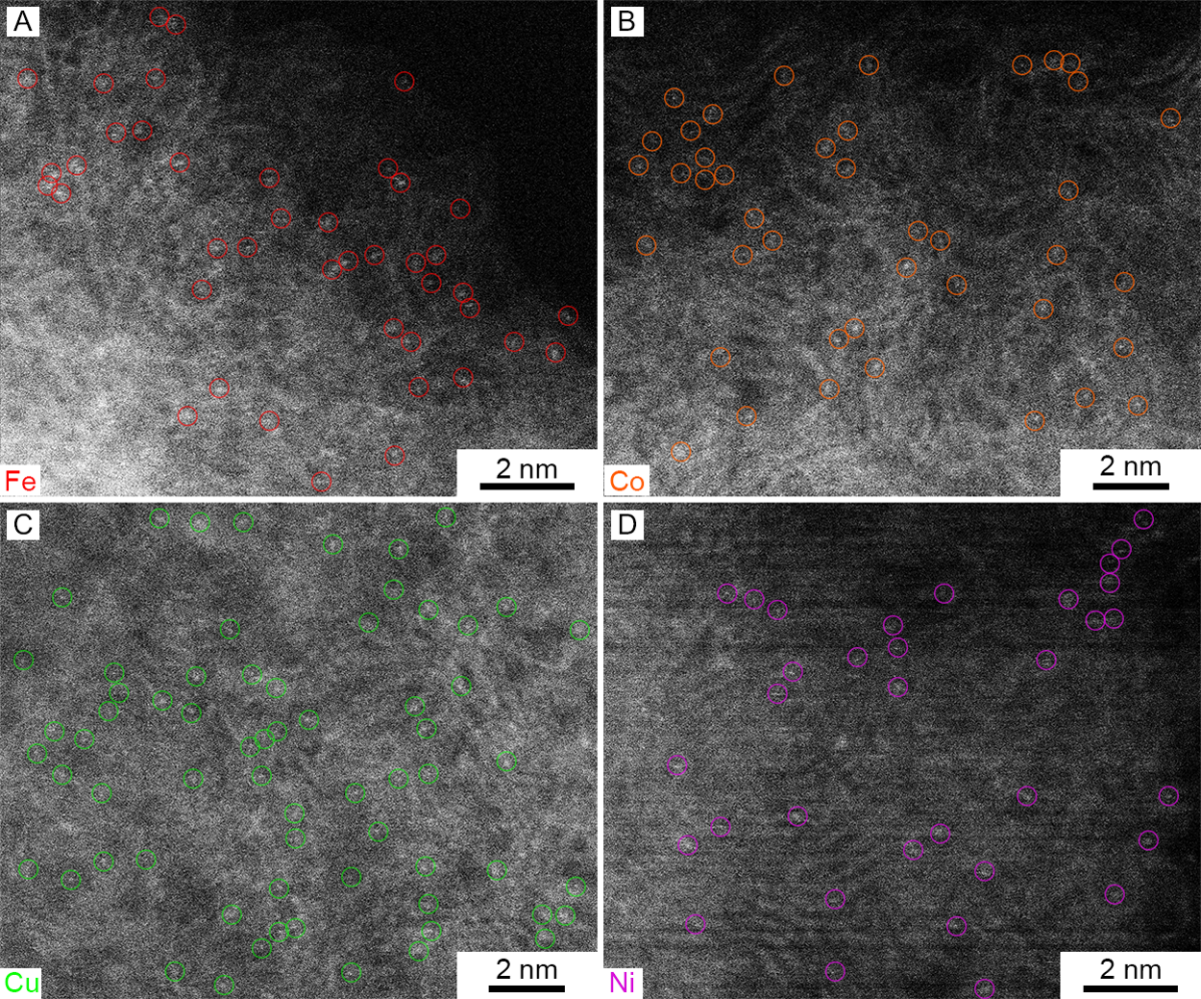
High-resolution HAADF-STEM images of (A) CS-Fe-E, (B) CS-Co-E, (C) CS-Cu-E, and (D) CS-Ni-E. Colored circles indicate the locations of some representative SACs.

**Figure 3:**
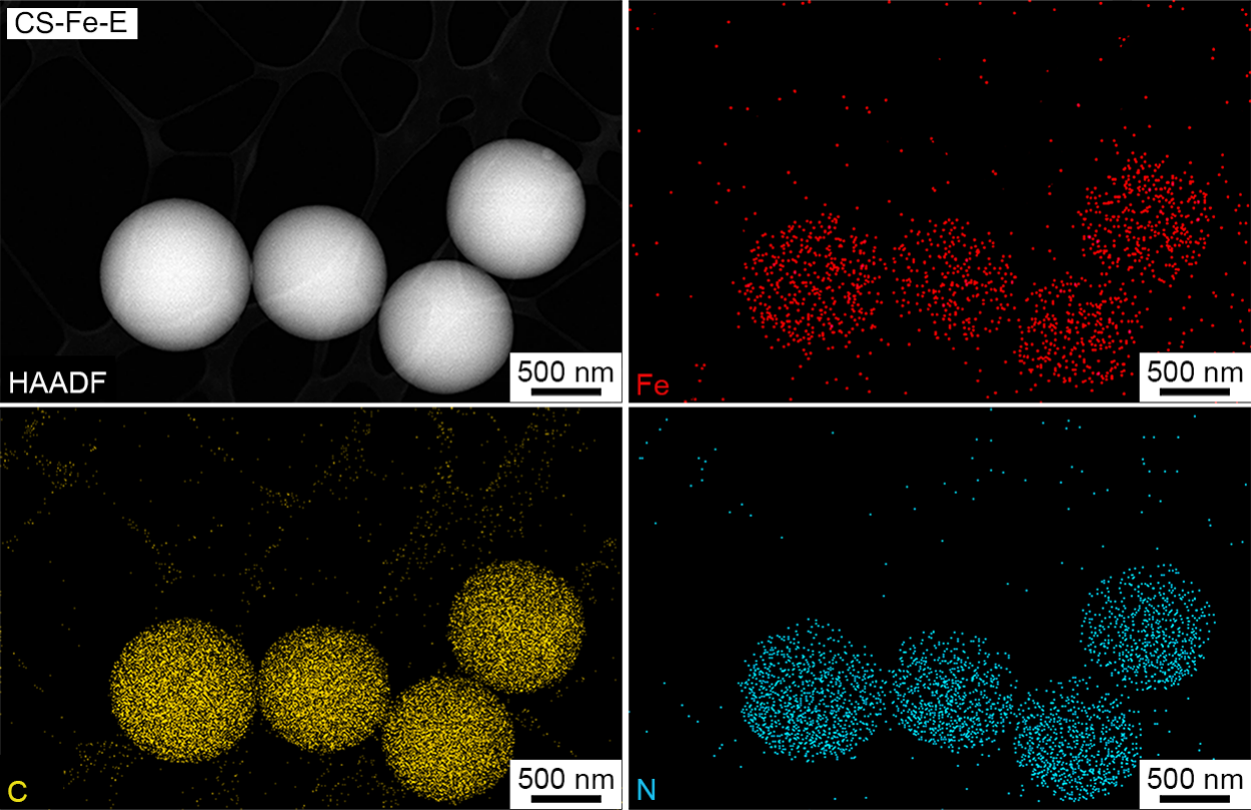
HAADF and the corresponding elemental mapping images of the CS-Fe-E particles.

Because this synthesis utilizes a chemical confinement strategy that is exclusively dependent on EDTA, the metal species can be readily changed to a wide variety of transition metals, making the synthesis versatile. Here we focus on four different transition metals, specifically, Fe, Co, Cu, and Ni, to have them incorporated into uniform carbon nanospheres at high loadings using a one-pot method. It is important to note that this synthesis does not involve any acid leaching steps, which not only demonstrates the effectiveness of EDTA in maintaining atomic isolation but also increases the practicality of the synthesis for industrial applications. The HAADF-STEM images in Figure 2 confirm that the transition metals exist solely as individual atoms. Additionally, low-magnification HAADF-STEM and EDS mapping data suggest that the metal atoms are uniformly dispersed throughout the carbon nanospheres (Figure 3 and Figures S1–S3).

**Figure 4:**
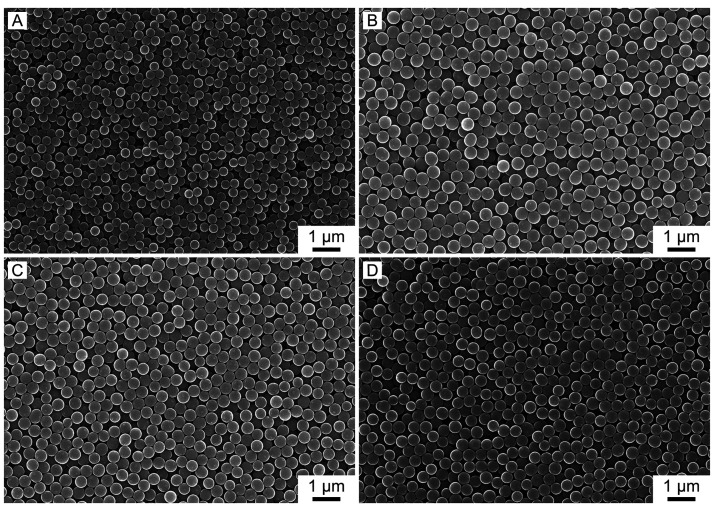
SEM images of the carbon nanospheres loaded with transition metal SACs: (A) CS-Fe-E, (B) CS-Co-E, (C) CS-Cu-E, and (D) CS-Ni-E.

Evidently, complexing EDTA with various transition metals had no impact on the spherical morphology, and thus highly uniform carbon nanospheres were obtained for all samples using the standard synthesis (Figure 4). The diameters of CS-Fe-E, CS-Co-E, CS-Cu-E, and CS-Ni-E were 421 ± 10, 466 ± 13, 464 ± 14, and 444 ± 25 nm, respectively, showing a thermal shrinkage of less than 25% compared to their polymer counterparts (Figure S4). The specific surface area and pore size distribution were measured by using N_2_ sorption analysis (Figure S5). All the samples exhibited a type II isotherm, indicating a nonporous surface. The specific surface areas of CS-Fe-E, CS-Co-E, CS-Cu-E, and CS-Ni-E were 11.1, 8.2, 8.34, and 6.85 m^2^ g^–1^, respectively. Raman spectroscopy was used to evaluate the degree of graphitization in the carbon structure (Figure S6). All of the samples displayed two peaks around 1350 and 1570 cm^–1^, corresponding to the D and G bands, respectively. The *I*_D_/*I*_G_ ratios (i.e., degree of graphitization) of CS-Fe-E, CS-Co-E, CS-Cu-E, and CS-Ni-E were 0.74, 0.66, 0.61, and 0.85, respectively. These ratios are quite low compared to samples prepared in our previous study without metal SACs, suggesting that the metal-EDTA precursor might have promoted catalytic graphitization which is beneficial to the improvement of the conductivity for electrocatalysis.^22^ Additionally, the absence of any sharp metal peaks in the fingerprint region of the Raman spectra indicated no presence of metal nanoparticles. The thermal stability and total metal content for each sample were measured using TGA (Figure S7). The initial decomposition temperatures for CS-Fe-E, CS-Co-E, CS-Cu-E, and CS-Ni-E were 380, 360, 450, and 300 °C, respectively. The deviation in thermal stability can be attributed to the differences in the degree of graphitization, as graphitic and turbostratic carbons are more thermally stable than amorphous carbon. Indeed, the degree of graphitization increased in the order of CS-Ni-E < CS-Fe-*E* < CS-Co-E < CS-Cu-E, which is similar to the increasing trend of thermal stability: CS-Ni-E < CS-Co-*E* ≈ CS-Fe-E < CS-Cu-E. The metal contents for CS-Fe-E, CS-Co-E, CS-Cu-E, and CS-Ni-E were found to be 1.82, 1.93, 3.87, and 2.03 wt %, respectively, which are some of the highest values reported for transition metal SACs in carbon nanospheres using a one-pot synthesis. Previously, Cao et al. developed a one-pot method by adding nickel acetylacetonate, Ni(acac)_2_, during the polymerization of dopamine, which produced Ni SACs at a loading of 1.85 wt % on carbon spheres. However, the preparation involved acid leaching, presumably to remove metal nanoparticles and the spherical morphology was largely destructed.^18^ Interestingly, they also attempted a synthesis with 3-aminophenol and formaldehyde monomers, but the SAC loading was dramatically reduced to 0.69 wt %. Likewise, many other researchers have attempted to increase the loading of SACs on carbon spheres using a one-pot synthesis but doing so without distorting the morphology remained elusive until this current work.^19,20,26^

To maximize the SAC loading, we optimized the amounts of M-EDTA and ammonia. Using Fe as an example, the amount of Fe-EDTA was changed from the standard synthesis at 150 mg to 300 mg, and the sample was denoted “CS-Fe-E-H”, where “H” refers to high loading. TGA indicated that the metal content was nearly doubled to 3.79 wt % for CS-Fe-E-H, but the spherical morphology was destroyed (Figure S8). The ammonia concentration was also found to have a major effect on the SAC loading and spherical morphology. Typically, ammonia serves dual functions as a catalyst for the polymerization reaction while also providing surface charges to the resultant droplets to coerce them into a spherical shape. However, it also appears to have a major impact on the formation of SACs and their loadings. In the absence of ammonia, the carbon support severely aggregated, which was expected as there was little driving force to prompt a spherical shape (Figure S9). In addition, we observed a large number of metal nanoparticles throughout the carbon structure. Alternatively, when the ammonia amount was increased to 0.4 mL, no nanoparticles were observed, and the structure was highly uniform. The metal loading was also significantly affected by the ammonia concentration. The sample prepared without ammonia contained nanoparticles and had a metal loading of 2.1 wt %, whereas the sample prepared with 0.4 mL of ammonia only gave a metal loading of 0.29 wt % (Figure S9). The formation of metal nanoparticles and the deviation in metal loading can be explained by the polymerization rate between 3-aminophenol and formaldehyde. In the absence of ammonia, polymerization occurred at a much slower rate, leading to an abundance of formaldehyde in solution to reduce Fe-EDTA and thus produce metal nanoparticles.^27^ In contrast, when ammonia was added, the polymerization occurred at a much faster rate, and therefore, the formaldehyde monomers were quickly depleted, eliminating the possibility for the reduction reaction to occur. However, if the polymerization rate was too fast, as observed in the sample with 0.4 mL NH_3_, Fe-EDTA might have difficulty to enter the droplets because the oligomers were cross-linked too rapidly, preventing Fe-EDTA from getting trapped in the polymer matrix and thus reducing the loading.

**Figure 5:**
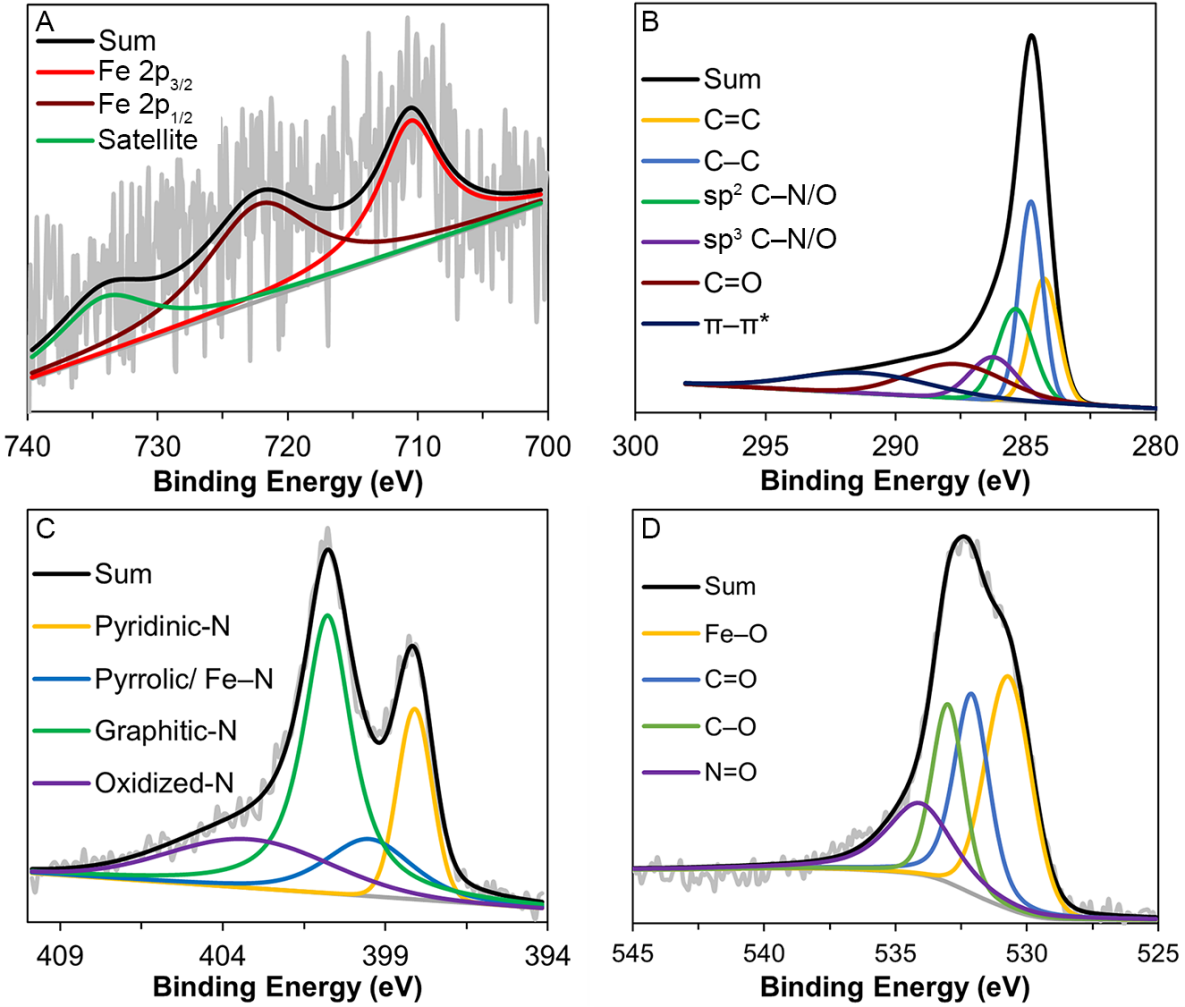
High-resolution XPS spectra of CS-Fe-E. (A) Fe 2p, (B) C 1s, (C) N 1s, and (D) the O 1s.

The chemical composition, oxidation state, and coordination environment of the samples were analyzed by using XPS and XAS. Deconvolution of the XPS spectra revealed that all samples had similar C, N, and O species (Figure 5 and Figures S10–S12). The high-resolution C 1s spectrum of CS-Fe-E was deconvoluted into five chemical peaks: C=C (284.28 eV), C–C (284.78 eV), sp^2^ C–N/O (285.38 eV), sp^3^ C–N/O (286.28 eV), and C=O (287.78 eV).^28−31^ Additionally, a broad π–π* shakeup peak (291.88 eV) was observed, indicating the presence of graphitic carbons.^32^ The high-resolution N 1s spectrum was deconvoluted into pyridinic-N (398.08 eV), pyrrolic/Fe–N (399.58 eV), graphitic-N (400.78 eV), and oxidized-N (403.48 eV) species.^22,33−35^ The high-resolution O 1s spectrum was deconvoluted into Fe–O (530.78 eV), C=O (532.08), C–O (532.98), and N=O (534.18 eV).^31,36^ Lastly, the high-resolution Fe 2p spectrum was deconvoluted into Fe 2p_3/2_ (710.48 eV), Fe 2p_1/2_ (721.68 eV), and a small Fe 2p_1/2_ satellite (733.38 eV), suggesting an oxidation state slightly lower than Fe^3+^.^36,37^ The XPS spectra for CS-Co-E, CS-Cu-E, and CS-Ni-E also revealed similar chemical compositions except that Co^2+^, Cu^2+^/Cu^+^, and Ni^2+^/Ni^+^ were observed, respectively (Figures S10–S12).^38−40^

**Figure 6:**
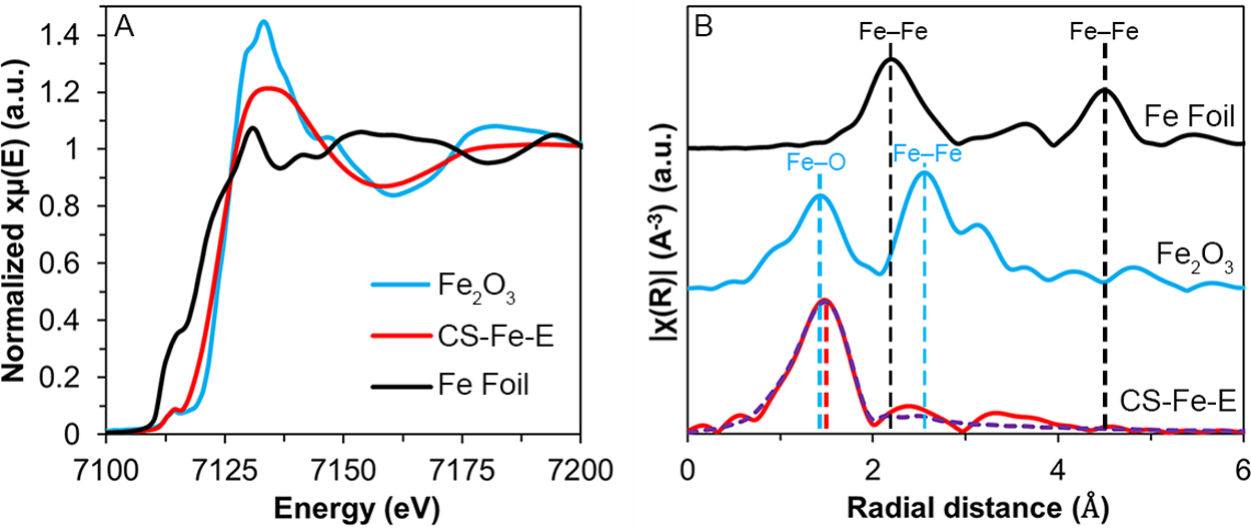
(A) Experimental XANES spectra of the Fe K-edge and (B) Fourier transform (FT) magnitudes of EXAFS spectra in the R space of CS-Fe-E. The purple dashed line represents the theoretical EXAFS fitting.

However, it is important to note that while the N 1s XPS spectrum may show metal–N bonds, their binding energies are located in the same range as other N species, and thus their existence is ill-defined. For this reason, XAS was conducted on the samples to elucidate the oxidation states and coordination environments of the Fe, Co, Cu, and Ni SACs. Based on the Fe K-edge X-ray absorption near edge structure (XANES) spectrum in Figure 6A, the average valence of Fe in CS-Fe-E was +2.52, which is consistent with the XPS data. The coordination environment evaluated by extended X-ray absorption fine structure (EXAFS) revealed that the Fe SACs had an average bond length of 1.98, corresponding to Fe–O and Fe–N moieties (Figure 6B). Moreover, the absence of any Fe–Fe peaks at 2.47 confirmed that the Fe species in CS-Fe-E were indeed atomically dispersed, which was consistent with the STEM data in Figure 2. EXAFS fitting was also performed on both R space and *k* space data to obtain structural parameters (Figure 6B and Figure S13A). Based on the best fitting EXAFS data, the coordination number (CN) of the Fe–N/O moieties was determined to be 4.3 (Table S1). Likewise, the XANES and EXAFS analyses of CS-Co-E, CS-Cu-E, and CS-Ni-E also indicated that the metals in each sample were atomically isolated and coordinated to nitrogen and oxygen with an average valence of +1.97, + 0.78, and +0.87, respectively (Figures S13–S16 and Tables S2–S4). Considering that the metal center in the initial M-EDTA complex was chelated to an abundance of nitrogen and oxygen groups, it makes sense that some of these bonds persisted during carbonization, leading to the formation of SACs that were partially bound to nitrogen and oxygen. This also explains why nanoparticles were not formed during the pyrolysis process. It is commonly reported that SACs tended to migrate at moderate carbonization temperatures and sinter into nanoparticles.^41−43^ However, this does not appear to be an issue in our case, presumably from the stubbornness of EDTA to remain partially bound to the metal ions during carbonization; essentially acting as a permanent anchor. Altogether, EDTA not only protects the metal centers from the initial reaction conditions but also stabilizes them during carbonization, enabling the production of SACs in high loadings without concern of nanoparticle formation. To further demonstrate the importance of EDTA, control samples were prepared using the same protocol except that Fe-EDTA was replaced with FeCl_3_ or Fe(acac)_3_. Due to the lack of stability under the alkaline reaction conditions, FeCl_3_ quickly precipitated during the synthesis, resulting in severe aggregation and formation of metal nanoparticles (Figure S17A). Interestingly, the spherical morphology was somewhat preserved when Fe(acac)_3_ was used, implying that Fe(acac)_3_ did not precipitate during the synthesis. Nevertheless, Fe(acac)_3_ was an insufficient molecular barrier and thus also produced metal nanoparticles (Figure S17B). These results confirmed the unique ability of EDTA to completely encapsulate metal ions and act as a molecular barrier to ensure atomic isolation.

**Figure 7:**
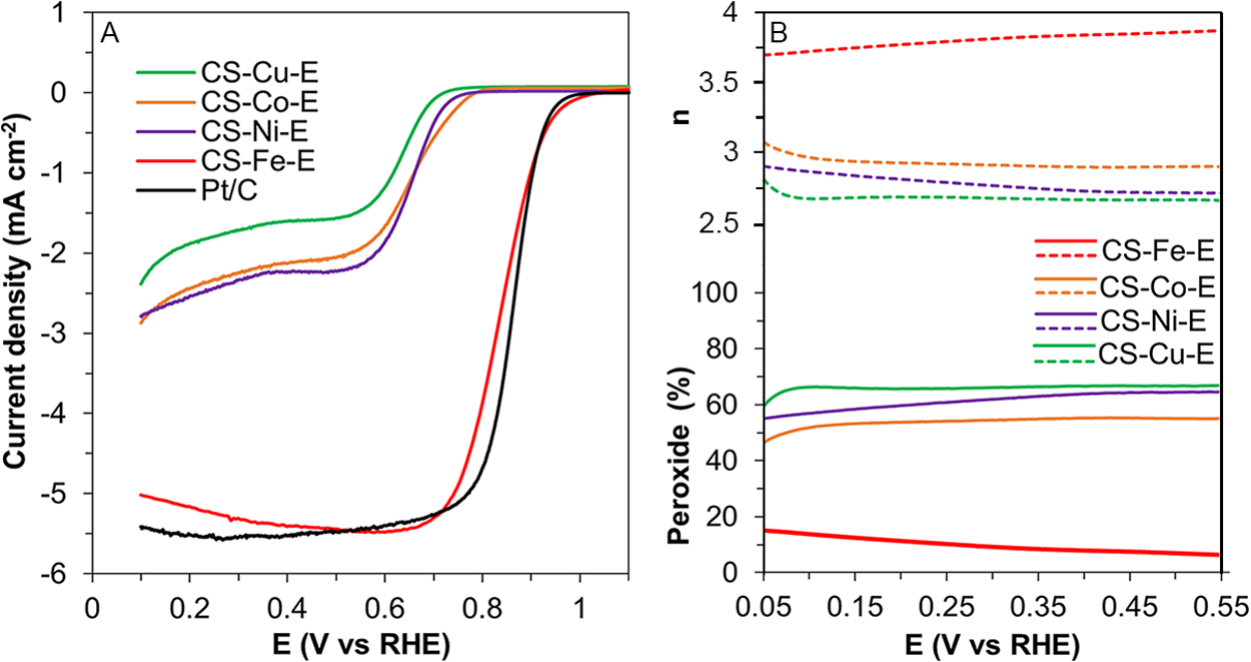
(A) Combination of positive sweeping ORR polarization curves recorded in O_2_-saturated 0.1 M KOH; (B) electron transfer number (top) and peroxide yield (bottom). All ORR polarization curves were *iR* and background corrected.

We evaluated the ORR electrocatalytic performance of the prepared catalysts in an O_2_-saturated 0.1 M KOH. As evident by the ORR polarization curves in Figure 7A, the sample containing Fe SACs exhibited a dramatic difference in activity compared to the samples containing Co, Cu, and Ni. This deviation in activity results from the different reaction pathways that are occurring during oxygen reduction. In aqueous solution, ORR primarily proceeds via two competing pathways: the direct 4e^–^ pathway, where dissolved oxygen is fully reduced to water, and the 2e^–^ pathway, where dissolved oxygen is only partially reduced to hydrogen peroxide. Though many factors influence reaction selectivity, a major factor is the type of adsorption between oxygen and catalytic sites. Yeager-type adsorption (i.e., side-on bridge coordination between two adjacent metal atoms) stabilizes each oxygen atom so that complete cleavage of O_2_ is possible, thus allowing both oxygen atoms to be completely reduced to water.^44^ Alternatively, Pauling-type adsorption (i.e., end-on coordination with a single metal atom) only stabilizes a single oxygen atom and thus O_2_ cleavage is prevented, causing an incomplete reduction to produce hydrogen peroxide.^44,45^ There is a lot of debate on whether SACs should be able to catalyze the 4e^–^ pathway because they lack vicinal metal atoms, so bridged O_2_ intermediates are not possible. Despite this, many reports have claimed that SACs, specifically Fe, are capable of catalyzing ORR via the 4e^–^ pathway. Interestingly, our data also supports this atypical selectivity trend. CS-Fe-E exhibited a max current density of 5.49 mA cm^–2^, with an onset (*E*_onset_) and half-wave (*E*_1/2_) potential of 1.00 V_RHE_ and 0.831 V_RHE_, respectively (Table S5). Remarkably, the *E*_onset_ and *E*_1/2_ of CS-Fe-E were very similar to 20 wt % Pt/C, which were 0.997 V_RHE_ and 0.843 V_RHE_, respectively. These results are even better than the recent reports of similar SAC-type catalysts (Table S6).^46−57^ The H_2_O_2_ yield and the electron transfer number (*n*) of CS-Fe-E were determined to be 7.87% and 3.84 at 0.4 V_RHE_, respectively, using the ring current density (*I*_r_) and eqs (Figure 7B and eqs S1 and S2). To investigate the O_2_ diffusion dependence and further confirm the *n* of Cs–Fe-E, LSV curves were acquired at various rotation rates between 400–2025 rpm (Figure S18A). The derived Koutecky–Levich (K–L) plots in Figure S18B were linear and parallel, indicating the ORR was first-order with respect to O_2_ and the average *n* calculated using the K–L eqs (eq S3–S4) was 3.61, confirming that CS-Fe-E indeed catalyzed ORR via the 4e^–^ pathway. Moreover, CS-Fe-E retained 93% of the initial ORR activity after 10 h of continuous usage, which was far better than that of Pt/C (Figure S19).

We also investigated the effects of carbonization temperature, loading, and metal precursor on ORR. As expected, the ORR performance increased with increasing carbonization temperature from 800 to 1000 °C, which could be attributed to a combination of increased conductivity and porosity (Figure S20A).^58^ However, it was also possible that the increased temperature was effectively regulating the SAC coordination environment to favor the 4e^–^ pathway.^59^ Interestingly, the CS-Fe-E-H sample also displayed a lower activity than the optimized CS-Fe-E sample. Despite having a higher loading, CS-Fe-E-H severely aggregated, as observed in Figure S8, which consequently blocked active sites and hindered oxygen diffusion, resulting in poor ORR performance. This result demonstrated the necessity of a highly uniform spherical morphology. To further illustrate the importance of EDTA, the samples prepared using other Fe precursors, specifically, FeCl_3_ and Fe(acac)_3_ were evaluated for ORR, and neither sample was better than CS-Fe-E (Figure S20B). Taken together, the high current density, low overpotential, and 4e^–^ selectivity make CS-Fe-E an ideal candidate to potentially replace the expensive Pt-based catalysts in energy production devices.

As is evident from the ORR polarization curves in Figure 7A, the samples containing Co, Cu, and Ni SACs showed a dramatic decrease in current density and a low *E*_onset_. Though it may appear that these samples had a low catalytic activity when compared to CS-Fe-E, the actual reason for the decreased values could be attributed to the selectivity shift from 4e^–^ to 2e^–^, meaning that the current density would be halved, which was consistent with our data (Table S5). Expectedly, the hydrogen peroxide yield increased to 55–67% for Co, Cu, and Ni samples, and the *n* values decreased to 2.66–2.89, indicating a preference toward the 2e^–^ pathway (Figure 7B). The deviation in activity between CS-Fe-E and the other samples can be explained by the difference in coordination environment. Many reports suggest a strong correlation between the coordination species and ORR performance. Specifically, SACs bound to nitrogen sites tend to be responsible for promoting the 4e^–^ pathway, whereas SACs bound to oxygen tend to promote the production of hydrogen peroxide through the 2e^–^ pathway.^60−62^ As such, it may be possible that the Fe SACs have a higher ratio of nitrogen coordination sites compared to the other samples, resulting in a selectivity shift toward the 4e^–^ pathway. Despite the XPS and XAS data that suggests that all SAC samples were bound to nitrogen and oxygen, EXAFS alone cannot be used to distinguish the ratios between nitrogen and oxygen coordination sites due to their similar atomic weights.^63^ Thus, we cannot quantify the ratio of N/O sites to confirm if this is responsible for the selectivity shift. Alternatively, it may be possible that O_2_ can bridge more readily between Fe–N/O bonds than other metal–N/O bonds, explaining why CS-Fe-E was more selective toward the 4e^–^ pathway. Interestingly, there seems to be a trend between the bond lengths of the SACs and their activity. For CS-Co-E, CS-Cu-E and CS-Ni-E, they all had similar bond lengths of 1.94–1.95 as determined by EXAFS, whereas CS-Fe-E had a notably longer bond length of 1.98 Å. Therefore, it may be possible that the longer bond length of Fe–N/O allows O_2_ to bind in a Yeager-type fashion and supports full reduction to water. Lastly, it is likely that the limited SAC exposure could be hindering ORR performance.^64,65^ The low surface area and lack of porosity observed in all CS-X-E samples inevitably prevent a large portion of SACs from being exposed for catalysis, reducing the ORR performance. In this regard, future work is needed to optimize the porosity. In any case, by simply changing the M-EDTA precursor, we could steer the ORR selectivity toward either energy generation or hydrogen peroxide production, making this synthesis highly desirable.

## Conclusions

In summary, we have demonstrated a versatile one-pot strategy to prepare transition metal SACs in highly uniform carbon nanospheres. The synthesis utilizes a chemical confinement strategy, where metal ions are first chelated to EDTA to form cage-like complexes that not only protect the metal ions from the alkaline conditions but also serve as a molecular barrier to spatially isolate the resultant metal atoms and thereby prevent them from aggregation. The synthesis is effective toward the production of a variety of transition metal SACs with remarkable loadings up to 3.87 wt % while maintaining a uniform, spherical morphology for the carbon support. Our ORR electrochemical studies further demonstrate that CS-Fe-E is highly selective toward the 4e^–^ pathway and exhibit a catalytic activity compatible to that of commercial 20 wt % Pt/C. Meanwhile, the Co, Cu, and Ni SACs show a major preference toward the 2e^–^ pathway, making them promising candidates for the electrochemical production of hydrogen peroxide.

## Supporting Information Available

HAADF-STEM, EDX mapping, TEM, N_2_ sorption analysis, Raman, TGA, XPS, XAS, and electrochemical results (PDF)
