# Supplementary material for: Single-Atom Catalysts for Selective Oxygen Reduction: Transition Metals in Uniform Carbon Nanospheres with High Loadings
Source: JACS Au. 2023 Oct 19;3(11):3227–36. doi: 10.1021/jacsau.3c00557 (PMC10685421; doi:10.1021/jacsau.3c00557)
Supplement: Supplementary file 1 — au3c00557_si_001.pdf [file au3c00557_si_001.pdf]

Supporting Information for

Single-Atom Catalysts for Selective Oxygen Reduction: Transition Metals in Uniform Carbon Nanospheres with High Loadings

Jacob Jeskey,[a] Yong Ding,[b] Yidan Chen,[b] Zachary D. Hood,[c] George E. Sterbinsky,[d] Mietek Jaroniec,[e] and Younan Xia[a,f,g]*

[a]School of Chemistry and Biochemistry, Georgia Institute of Technology, Atlanta, Georgia 30332, United States

[b]School of Materials Science and Engineering, Georgia Institute of Technology, Atlanta, Georgia 30332, United States

[c]Applied Materials Division, Argonne National Laboratory, Lemont, Illinois 60439, United States

[d]Advanced Photon Source, Argonne National Laboratory, Lemont, Illinois 60439, United States

[e]Department of Chemistry and Biochemistry, Kent State University, Kent, Ohio 44242, United States

[f]The Wallace H. Coulter Department of Biomedical Engineering, Georgia Institute of Technology and Emory University, Atlanta, Georgia 30332, United States

[g]School of Chemical and Biomolecular Engineering, Georgia Institute of Technology, Atlanta, Georgia 30332, United States

*Corresponding author: younan.xia@bme.gatech.edu

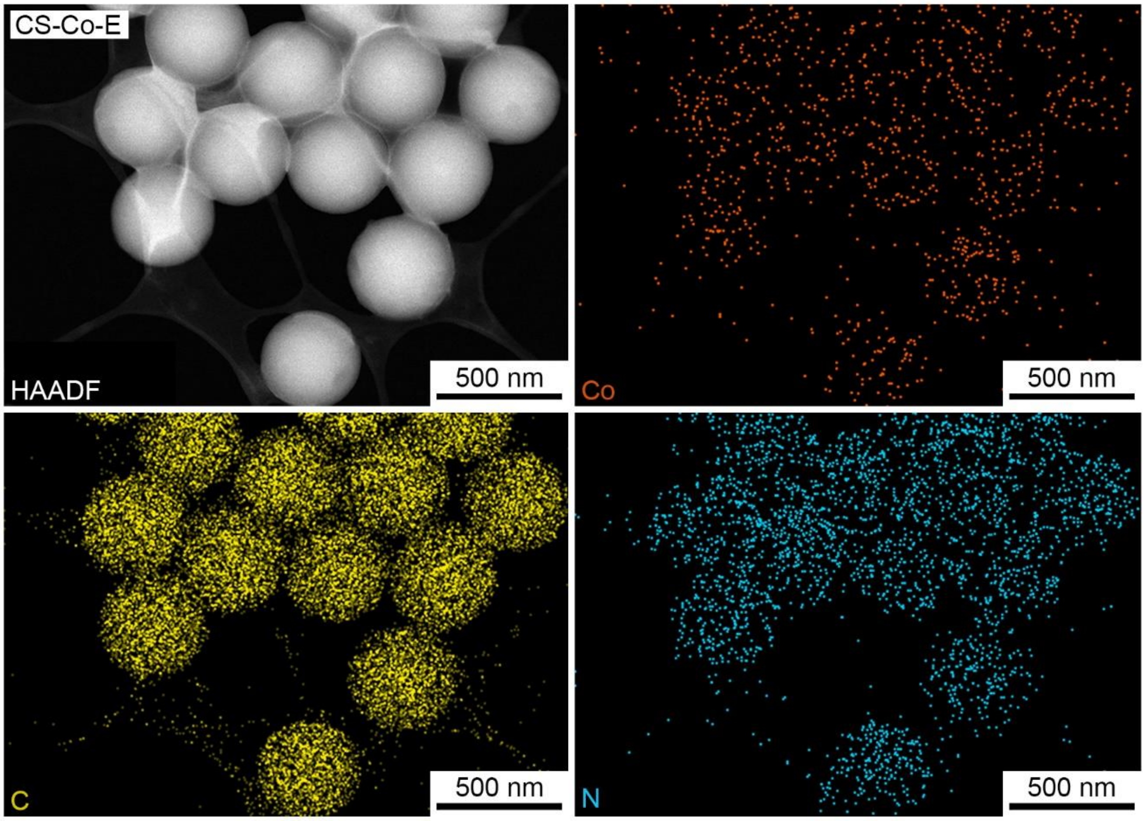

Figure S1. HAADF and the corresponding elemental mapping images of CS-Co-E.

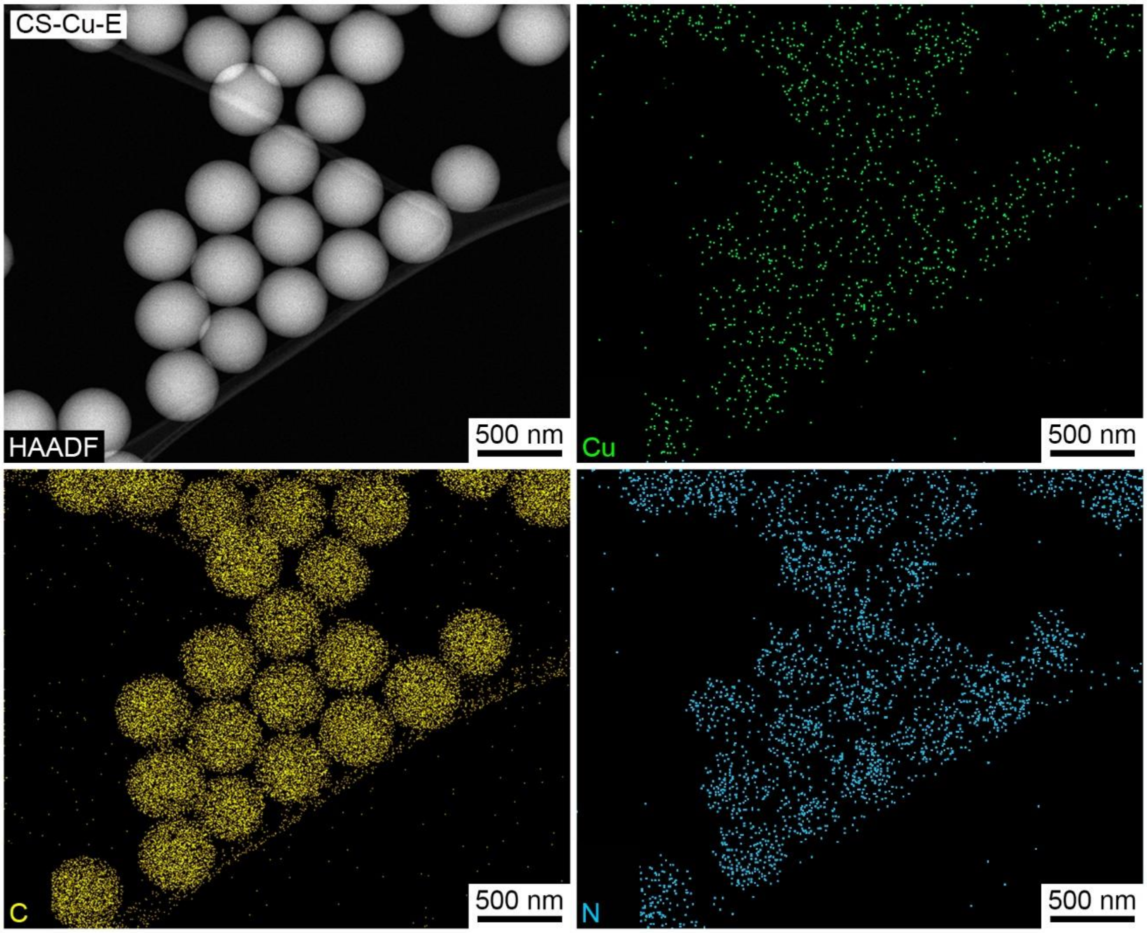

Figure S2. HAADF and the corresponding elemental mapping images of CS-Cu-E

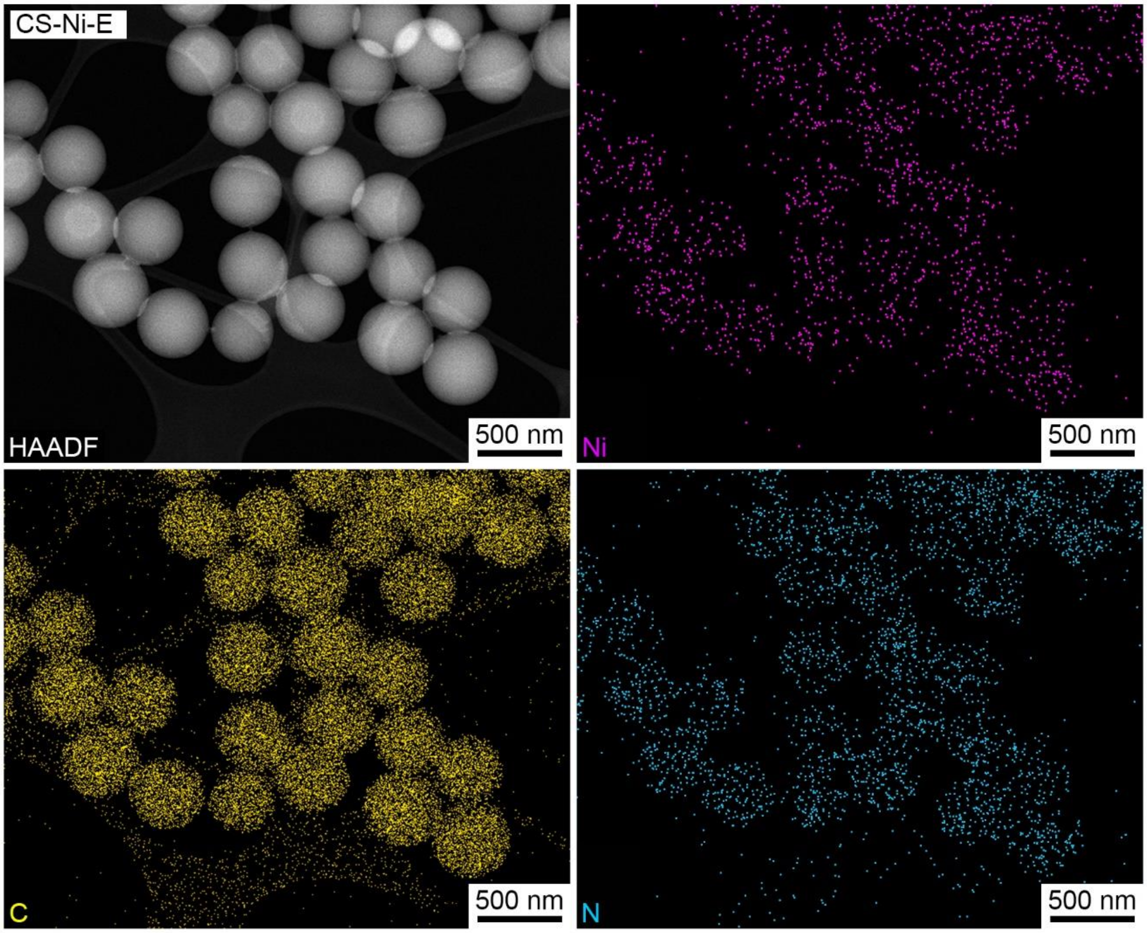

Figure S3. HAADF and the corresponding elemental mapping images of CS-Ni-E.

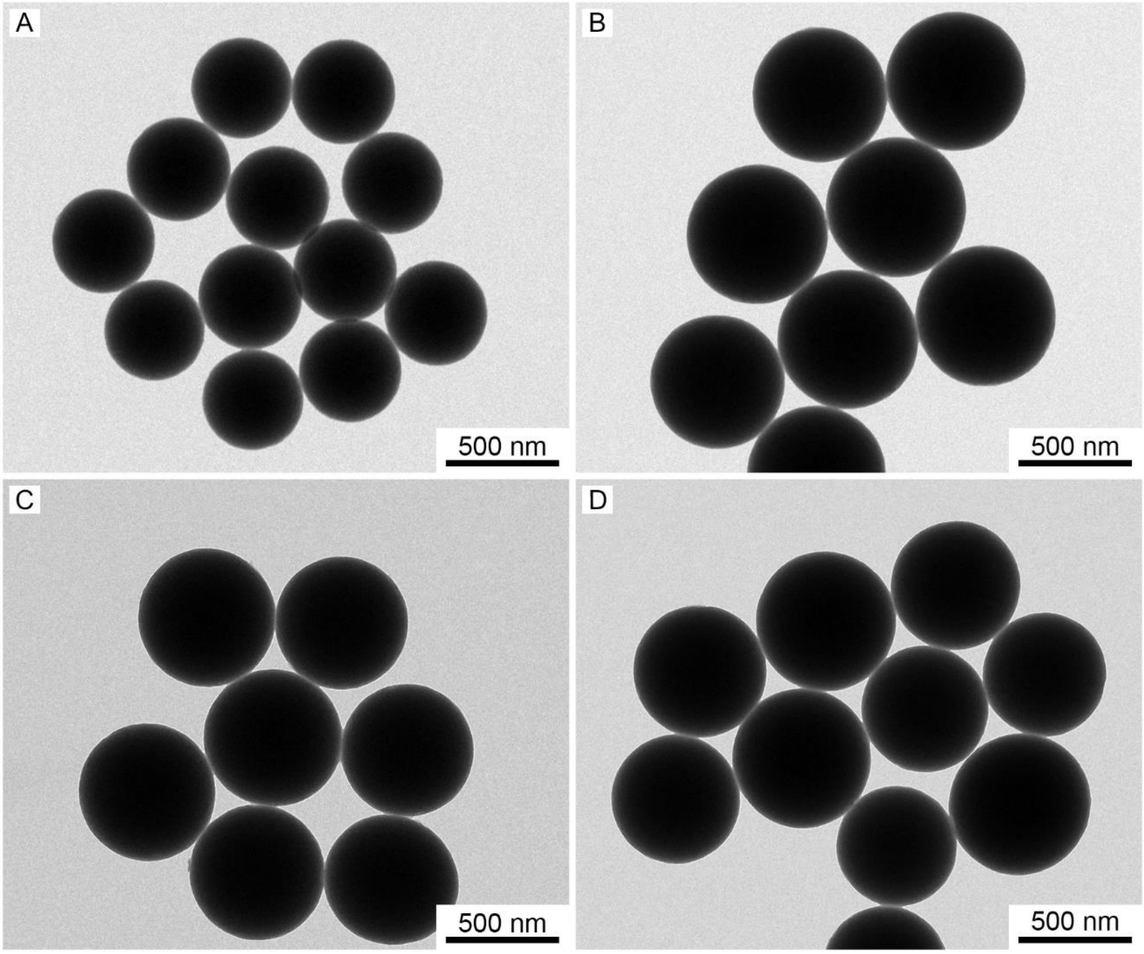

Figure S4. TEM images of the polymer nanospheres before carbonization. (A) PS-Fe-E, (B) PS-Co-E, (C) PS-Cu-E, and (D) PS-Ni-E. The sizes of the polymer nanospheres before carbonization were 454 ± 3, 611 ± 7, 592 ± 13, and 579 ± 21, respectively, for PS-Fe-E, PS-Co-E, PS-Cu-E, and PS-Ni-E.

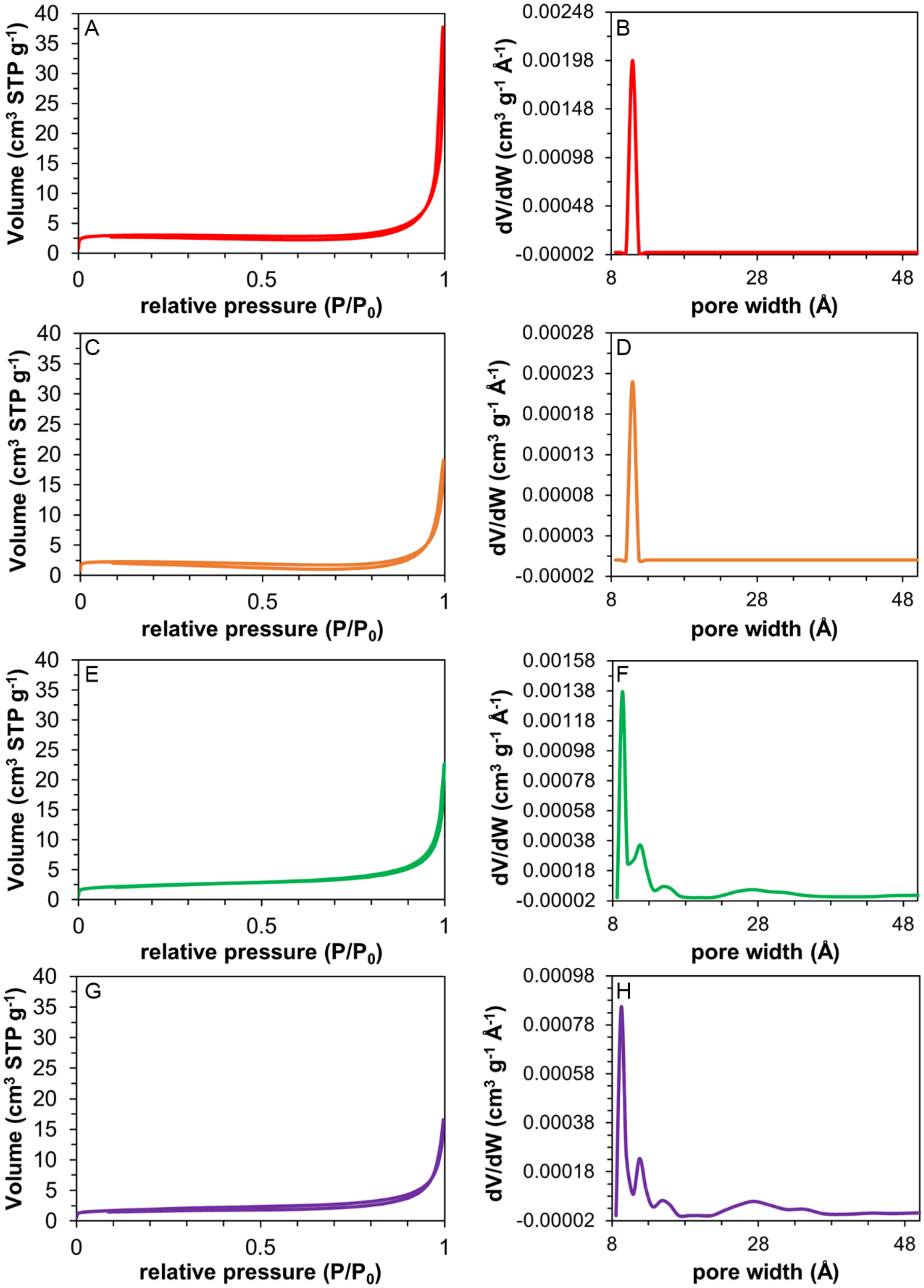

Figure S5. Nitrogen adsorption-desorption isotherms and corresponding pore size distribution curves for (A, B) CS-Fe-E, (C, D) CS-Co-E, (E, F) CS-Cu-E, and (G, H) CS-Ni-E.

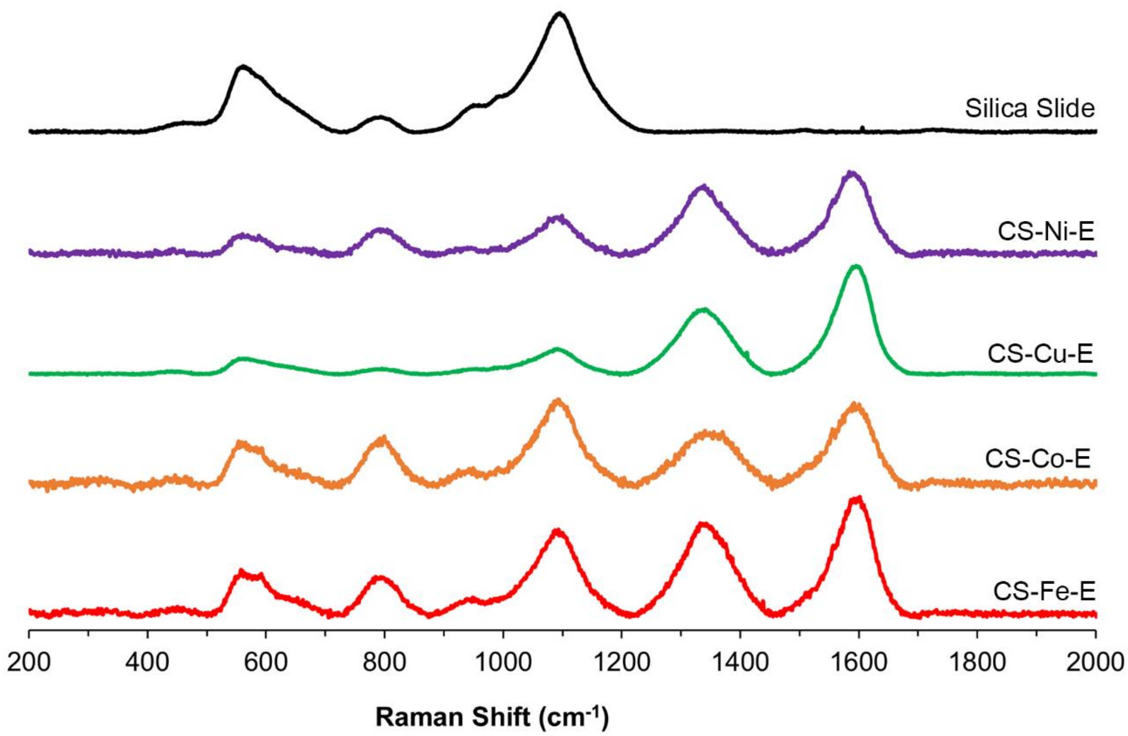

Figure S6. Comparison of Raman spectra for the samples studied. For reference, a blank silica slide was included to show that the three small peaks below 1000 cm^{-1} are a consequence of the silica sample holder and not due to metal nanoparticles.

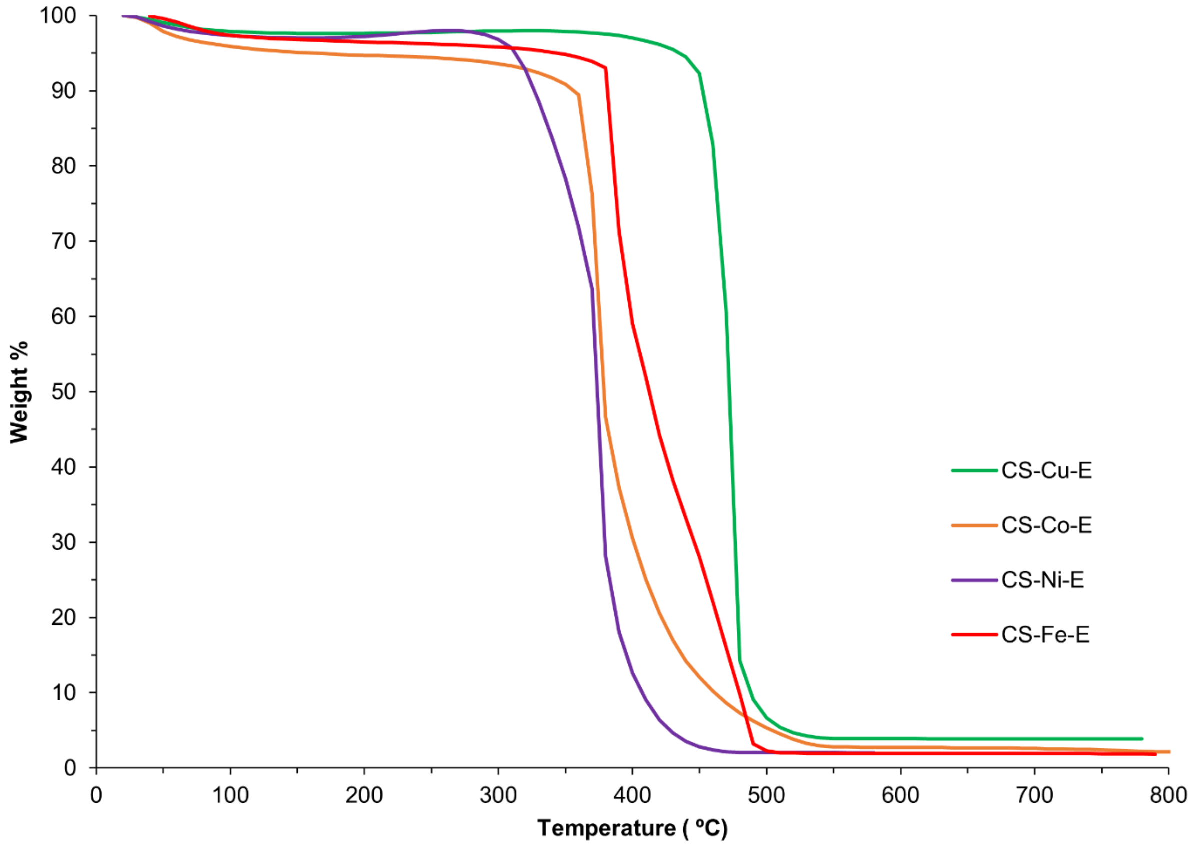

Figure S7. Thermogravimetric profiles of the samples studied. Final wt.% indicated that the metal content for each sample was: 1.82 for CS-Fe-E, 1.93 for CS-Co-E, 3.87 for CS-Cu-E, and 2.03 for CS-Ni-E.

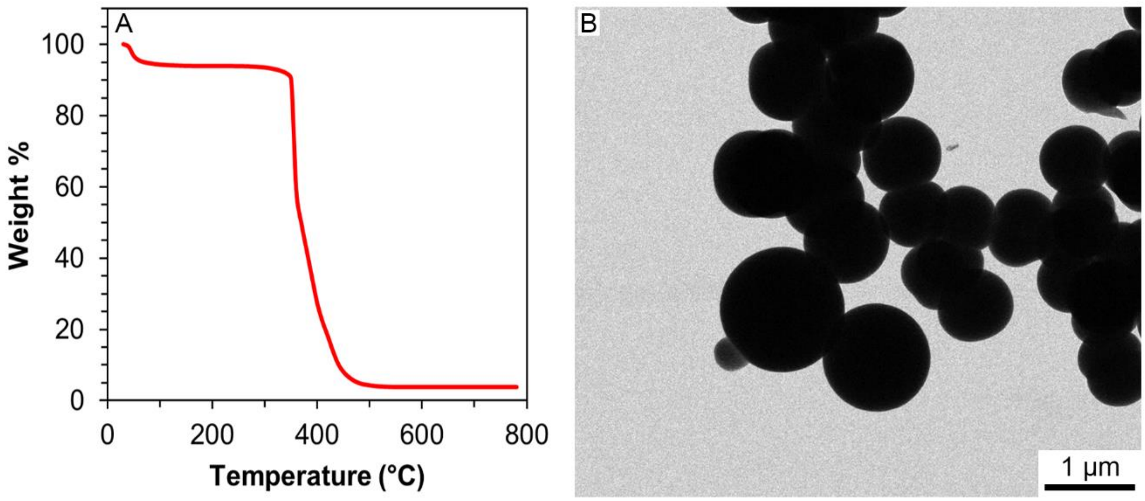

Figure S8. (A) Thermogravimetric profile and (B) TEM image of CS-Fe-E-H. Total metal content of CS-Fe-E-H was determined to be 3.79 wt.%.

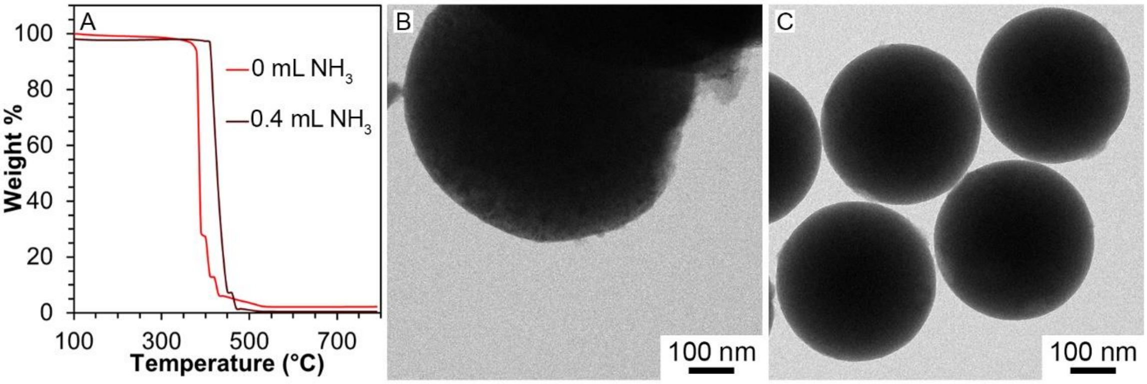

Figure S9. (A) Thermogravimetric profiles and TEM images of CS-Fe-E prepared using (B) 0 mL NH_3 and (C) 0.4 mL NH_3. Total metal contents were determined to be 2.10 wt.% and 0.29 wt.% when 0 mL and 0.4 mL of NH_3 was added, respectively.

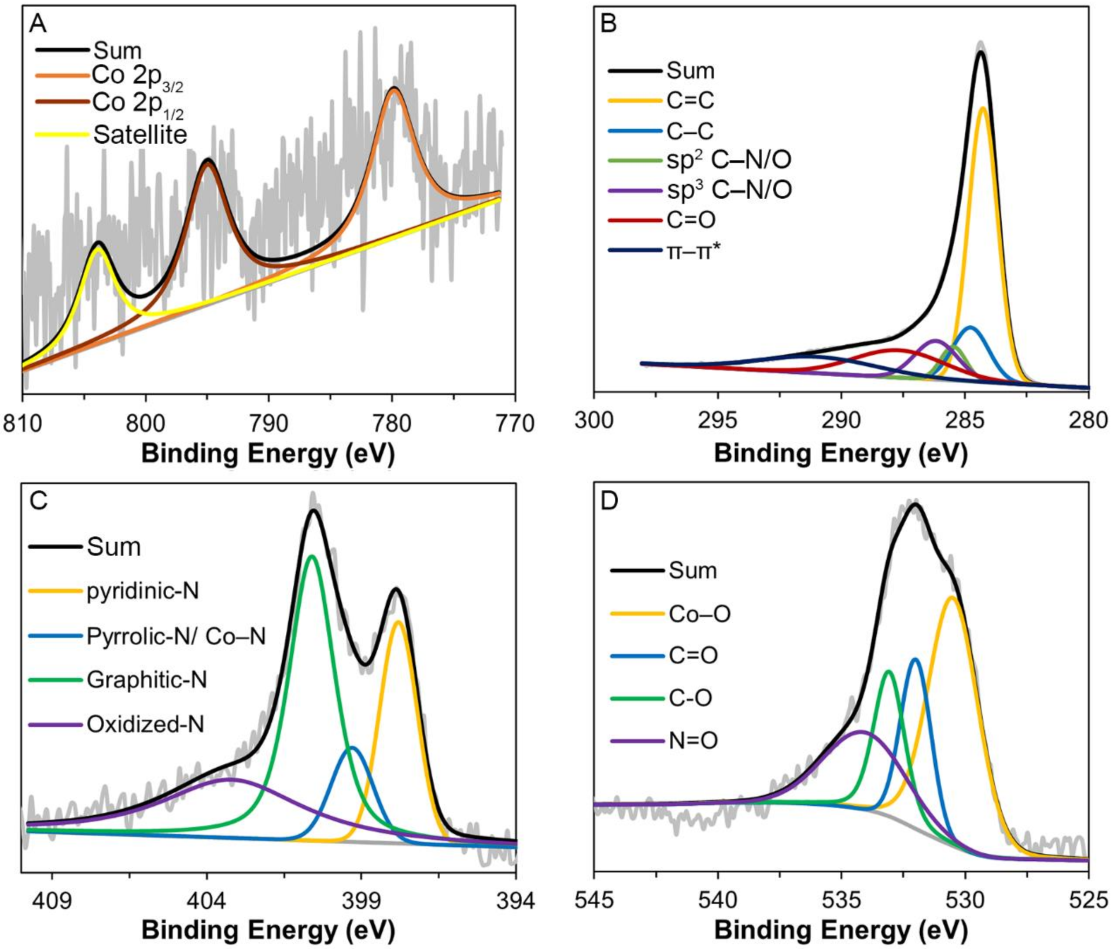

Figure S10. High-resolution XPS spectra of CS-Co-E. (A) Co 2p, (B) C 1s, (C) N 1s, and (D) O 1s.

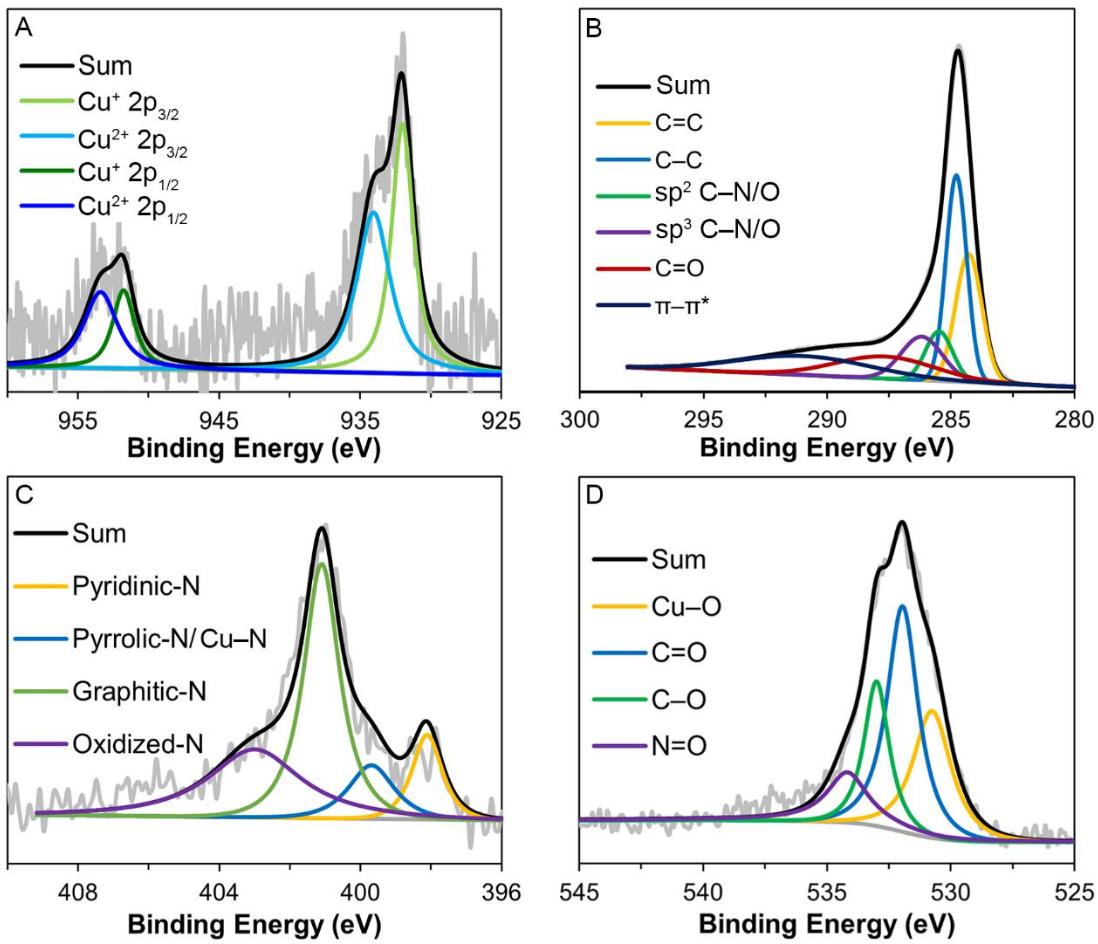

Figure S11. High-resolution XPS spectra of CS-Cu-E. (A) Cu 2p, (B) C 1s, (C) N 1s, and (D) O 1s.

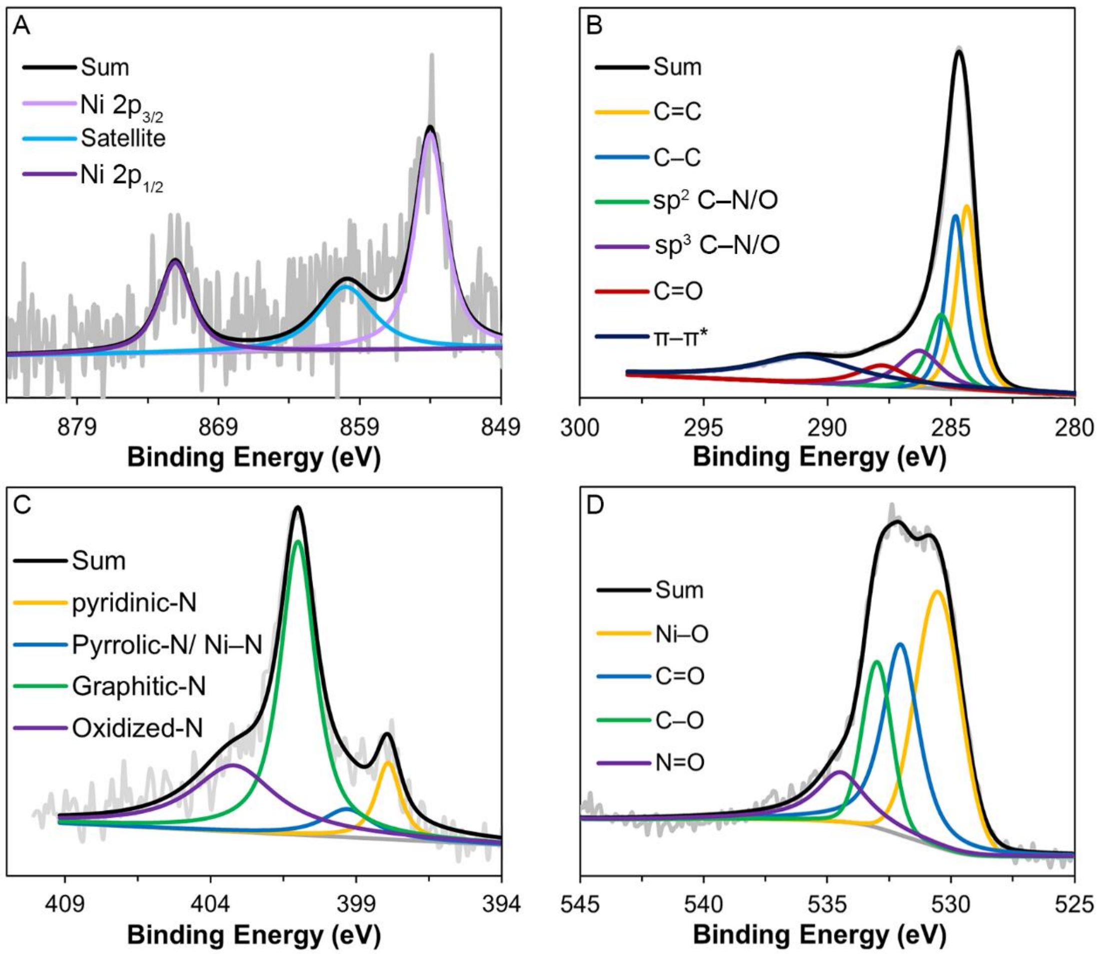

Figure S12. High-resolution XPS spectra of CS-Ni-E. (A) Ni 2p, (B) C 1s, (C) N 1s, and (D) O 1s.

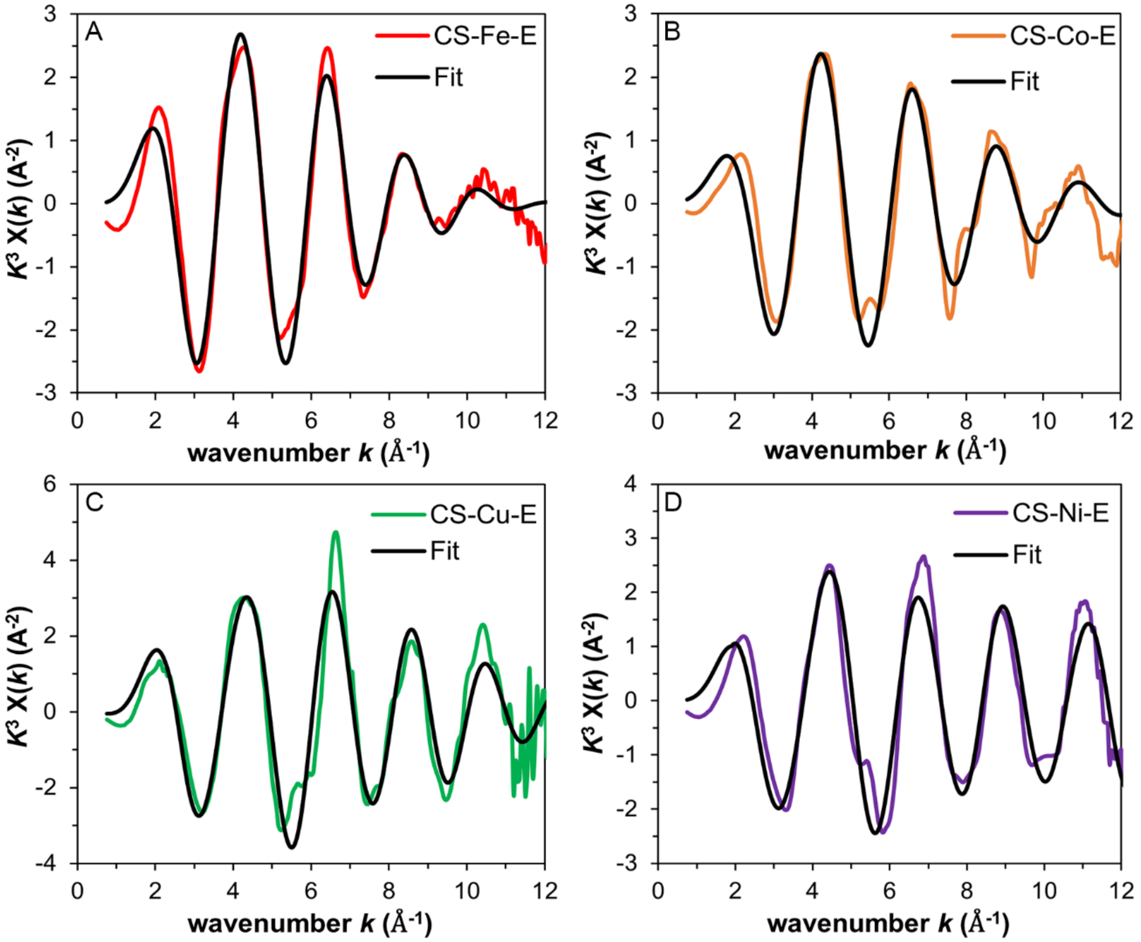

Figure S13. Experimental EXAFS spectra in *k* space with best fitting curve for (A) CS-Fe-E, (B) CS-Co-E, (C) CS-Cu-E, and (D) CS-Ni-E.

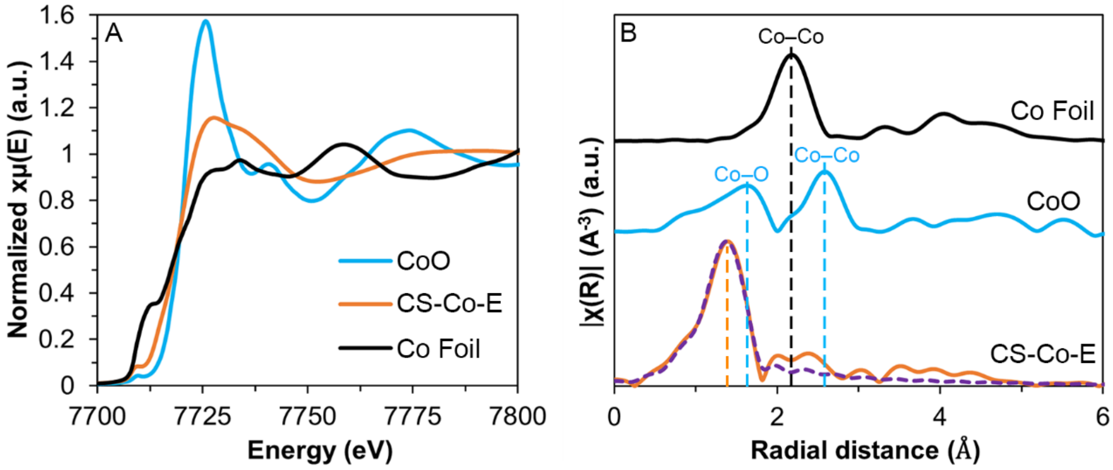

Figure S14. (A) Experimental XANES spectra of Co K-edge and (B) Fourier transform (FT) magnitudes of EXAFS spectra in R space of CS-Co-E. The purple dashed line represents the theoretical EXAFS fitting.

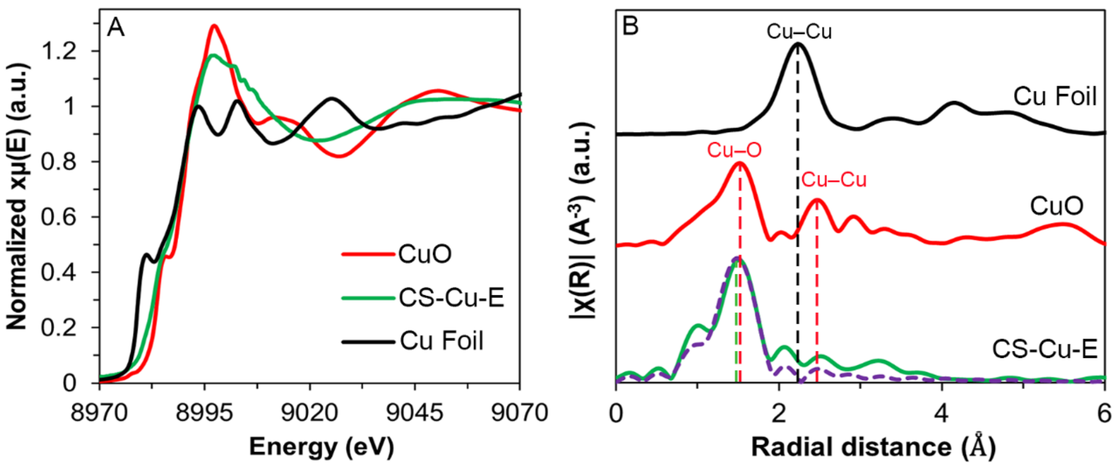

Figure S15. (A) Experimental XANES spectra of Cu K-edge and (B) Fourier transform (FT) magnitudes of EXAFS spectra in R space of CS-Cu-E. The purple dashed line represents the theoretical EXAFS fitting.

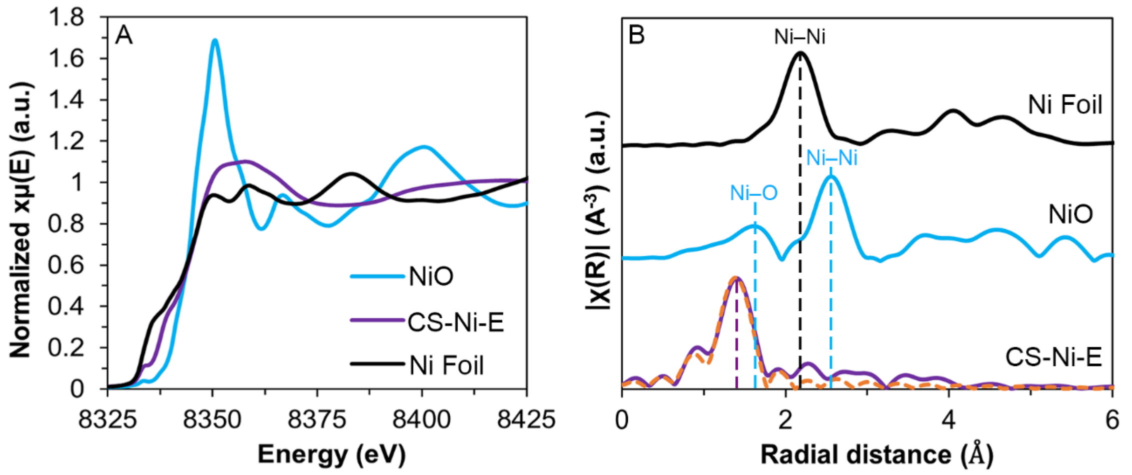

Figure S16. (A) Experimental XANES spectra of Ni K-edge and (B) Fourier transform (FT) magnitudes of EXAFS spectra in R space of CS-Ni-E. The orange dashed line represents the theoretical EXAFS fitting.

Table S1. EXAFS fitting parameters at the Fe K-edge for various samples (S_0^2=0.830).

Sample	Shell	N^a	R(Å)^b	σ^2(Å^2)^c	ΔE_0 (eV)^d	R factor
**Fe foil**	Fe–Fe	8	2.47	0.0056	6.3	0.0044
	Fe–Fe	6	2.84	0.0069	5.3	
**CS-Fe-E**	Fe–N/O	4.3	1.98	0.0070	-3.8	0.0002

$^a N$: coordination numbers; $^b R$: bond distance; $^c \sigma^2$: Debye-Waller factors; $^d \Delta E_0$: the inner potential correction. R factor: goodness of fit. S_0^2 was set to 0.830, according to the experimental EXAFS fit of Fe foil reference by fixing coordination numbers as the known crystallographic value. The k range and R range were set to 3.0–11.5 Å^{-1} and 1.0–3.0 Å, respectively.

Table S2. EXAFS fitting parameters at the Co K-edge for various samples (S_0^2=0.840).

Sample	Shell	N^a	R(Å)^b	σ^2(Å^2)^c	ΔE_0 (eV)^d	R factor
**Co foil**	Co–Co	12	2.49	0.0072	7.5	0.0022
**CS-Co-E**	Co–N/O	4.1	1.94	0.0097	-7.7	0.0016

$^a N$: coordination numbers; $^b R$: bond distance; $^c \sigma^2$: Debye-Waller factors; $^d \Delta E_0$: the inner potential correction. R factor: goodness of fit. S_0^2 was set to 0.840, according to the experimental EXAFS fit of Co foil reference by fixing coordination numbers as the known crystallographic value. The k range and R range were set to 3.0–11.5 Å^{-1} and 1.0–3.0 Å, respectively.

Table S3. EXAFS fitting parameters at the Cu K-edge for various samples (S_0^2=0.850).

Sample	Shell	N^a	R(Å)^b	σ^2(Å^2)^c	ΔE_0 (eV)^d	R factor
**Cu foil**	Cu–Cu	12	2.54	0.0086	4.1	0.0020
**CS-Cu-E**	Cu–N/O	4.2	1.95	0.0022	-2.5	0.0043

$^a N$: coordination numbers; $^b R$: bond distance; $^c \sigma^2$: Debye-Waller factors; $^d \Delta E_0$: the inner potential correction. R factor: goodness of fit. S_0^2 was set to 0.850, according to the experimental EXAFS fit of Cu foil reference by fixing coordination numbers as the known crystallographic value. The k range and R range were set to 3.0–12.0 Å^{-1} and 1.0–3.0 Å, respectively.

Table S4. EXAFS fitting parameters at the Ni K-edge for various samples (S_0^2=0.860).

Sample	Shell	N^a	R(Å)^b	σ^2(Å^2)^c	ΔE_0 (eV)^d	R factor
**Ni foil**	Ni–Ni	12	2.48	0.0066	6.6	0.0016
**CS-Ni-E**	Ni–N/O	4.2	1.94	0.0095	-8.2	0.0047

$^a N$: coordination numbers; $^b R$: bond distance; $^c \sigma^2$: Debye-Waller factors; $^d \Delta E_0$: the inner potential correction. R factor: goodness of fit. S_0^2 was set to 0.860, according to the experimental EXAFS fit of Ni foil reference by fixing coordination numbers as the known crystallographic value. The k range and R range were set to 3.0–12.0 Å^{-1} and 1.0–3.0 Å, respectively.

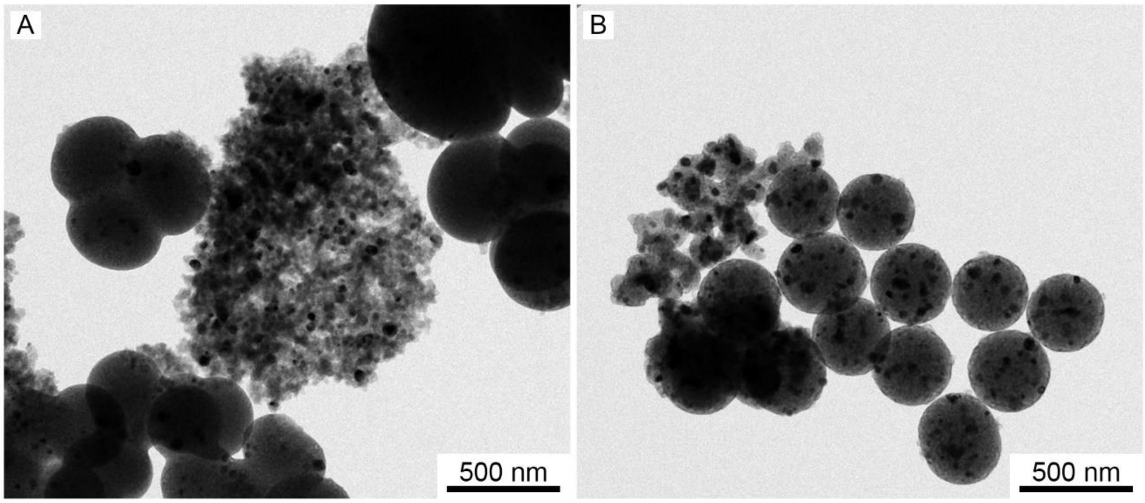

Figure S17. TEM images of control samples prepared using (A) $FeCl_3$ and (B) $Fe(acac)_3$.

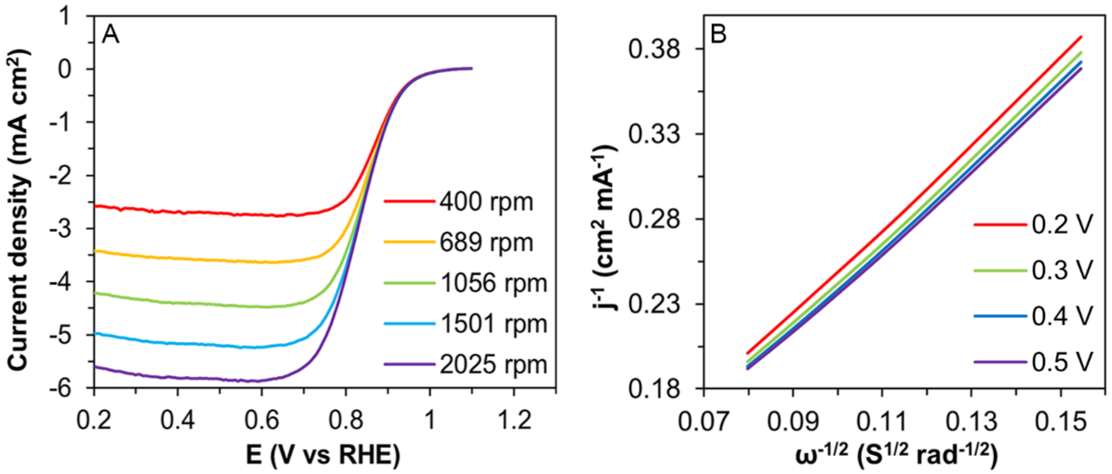

Figure S18. CS-Fe-E (A) LSV curves recorded at various rotation rates in O_2-saturated 0.1 M KOH and (B) corresponding K–L plots calculated at various potentials. All ORR polarization curves have been *iR* and background corrected.

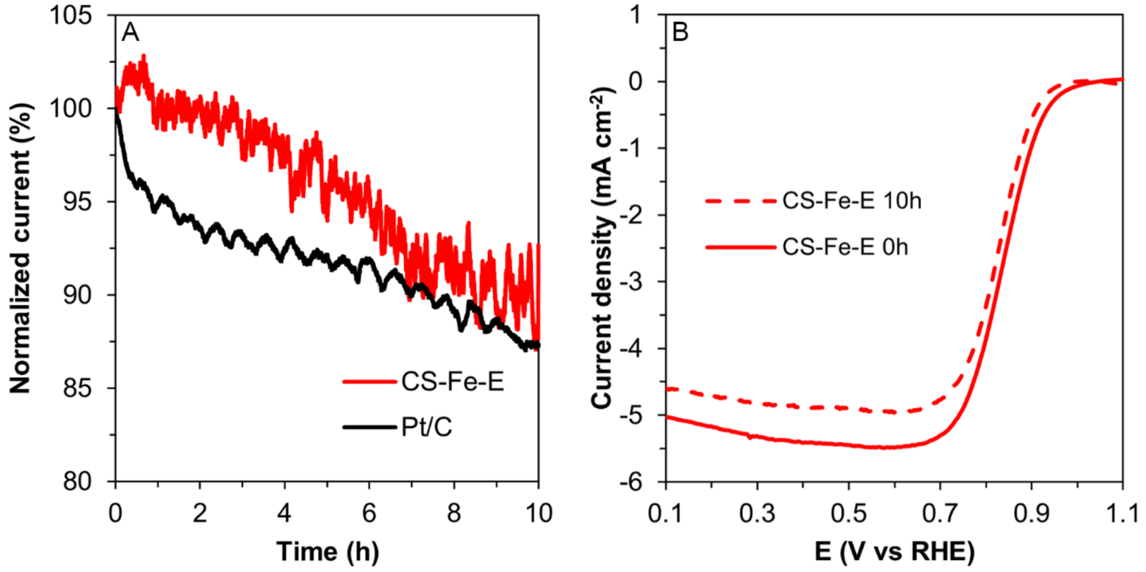

Figure S19. (A) Normalized i–t chronoamperometry curves at 0.5 V_{RHE} in O_2-saturated 0.1 M KOH at 1600 rpm and (B) Positive sweeping ORR polarization curves recorded before and after 10 h durability test. All data was *iR*-compensated at 85%. All ORR polarization curves have been iR and background corrected.

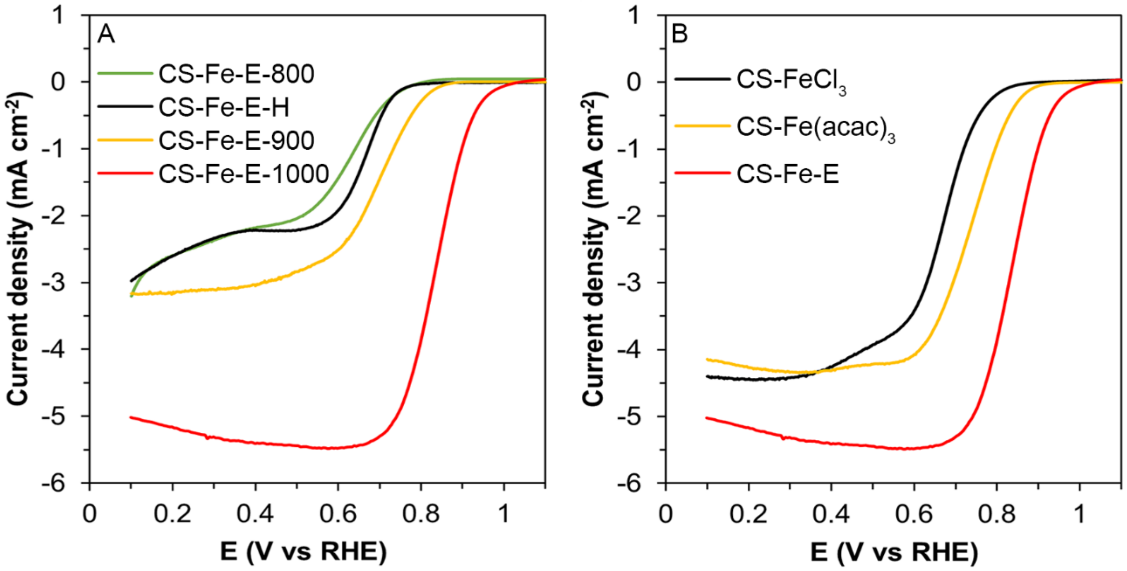

Figure S20. Combination of positive sweeping ORR polarization curves recorded in O_2-saturated 0.1 M KOH for (A) CS-Fe-E at different carbonization temperatures and loadings and (B) samples prepared with various Fe precursors. All ORR polarization curves have been *iR* and background corrected.

The H_2O_2 yield and electron transfer number for all samples was calculated using the following equations:

$$H_2O_2\% = \frac{200\frac{I_R}{N}}{(\frac{I_R}{N}+I_D)} \quad \text{Eq S1}$$

$$n = \frac{4I_D}{(\frac{I_R}{N}+I_D)} \quad \text{Eq S2}$$

Where I_R is the ring current, I_D is the disk current, N is the electron collection efficiency (25.6 %), and *n* is the electron transfer number.

The reaction order and electron transfer number for CS-Fe-E was calculated using the following equations:

$$\frac{1}{j} = \frac{1}{\beta\omega^{\frac{1}{2}}} + \frac{1}{j_K} \quad \text{Eq S3}$$

$$\beta = 0.62nFD_0^{\frac{2}{3}}v^{\frac{-1}{6}}C_0 \qquad \text{Eq S4}$$

Where j and j_K are the measured current density and the kinetic current density, respectively; ω is the electrode rotation speed; F is the Faraday constant (96485 C mol^{-1}); D_0 is the diffusion coefficient of O_2 (1.9 x 10^{-5} cm^2 s^{-1} for 0.1 M KOH; ν is the kinetic viscosity (0.01 cm^2 s^{-1}); and C_0 is the concentration of O_2 (1.2 x 10^{-6} mol cm^{-3}).

Table S5. ORR current density, onset potential and selectivity values for the samples studied. The electron transfer number was determined using ring current density (I_r) and equations S1–S2.

Sample	Current density (mA cm^{-1})	E_{onset} (V_{RHE})	$E_{1/2}$ (V_{RHE})	H_2O_2 %	*n* (0.4 V_{RHE})
CS-Fe-E	5.49	1.00	0.831	7.87	3.84
CS-Co-E	2.87	0.807	0.650	55.2	2.89
CS-Cu-E	2.39	0.734	0.641	66.7	2.66
CS-Ni-E	2.79	0.771	0.652	63.8	2.72

Table S6. Comparison of recently reported data for the ORR performance of SAC-type catalysts.

Catalyst	Electrolyte	Metal Content (wt. %)	E_{onset} (V_{RHE})	$E_{1/2}$ (V_{RHE})	Synthesis method	Reference
CS-Fe-E	0.1 M KOH	1.82	1.00	0.831	One-pot	This work
Pt-MnN_4	0.1 M KOH	5.09	0.95	0.88	One-pot	(46)
Co-N/C	0.1 M KOH	0.44 at.%	0.95	0.84	One-pot	(47)
1%Pt@C_4N	0.1 M KOH	0.31	0.923	0.861	One-pot	(48)
0.5%Ru@C_4N	0.1 M KOH	0.36	0.919	0.828	One-pot	(48)
Ru-N/G-750	0.1 M $HClO_4$	1.7	0.89	0.75	Post-mod	(49)
SAFe@NG	0.1 M KOH	4.6	0.96	0.876	Post-mod	(50)
Fe–Nx–CNF	0.1 M KOH	2.04	N/A	0.875	Post-mod	(51)
Fe SAs-Fe2P NPs/NPCFs-2.5	0.1 M KOH	2.5	1.03	0.91	Post-mod	(52)
C-FeHZ8@g-C3N4-950	0.1 M KOH	3.17	0.97	0.845	Hard template	(53)
2.0 wt % Cu-N-C SAC	0.1 M KOH	2.0	0.92	0.83	Hard template	(54)
2.0 wt % Co-N-C	0.1 M KOH	2.0	0.93	0.833	Hard template	(54)
2.0 wt % Fe-N-C	0.1 M KOH	2.0	0.91	0.803	Hard template	(54)
Co-NCS-2	0.1 M KOH	12.40*	0.96	0.90	Hard template	(55)
Fe-SAs/PNCH	0.1 M KOH	N/A	0.97	0.87	IWI	(56)
Ni-SAs/PNCH	0.1 M KOH	N/A	0.96	0.81	IWI	(56)
Fe@CNx	0.1 M KOH	9*	0.94	0.84	Ball milling	(57)
Mn@CNx	0.1 M KOH	6*	0.94	0.84	Ball milling	(57)
Co@CN_x	0.1 M KOH	17*	0.92	0.79	Ball milling	(57)
Cu@CNx	0.1 M KOH	14*	0.90	0.79	Ball milling	(57)
Ni@CNx	0.1 M KOH	11*	0.85	0.74	Ball milling	(57)
